# Composition considerations for fluid teams: a review

**DOI:** 10.3389/fpsyg.2024.1302022

**Published:** 2024-02-12

**Authors:** Tripp Driskell, Gregory Funke, Michael Tolston, August Capiola, James Driskell

**Affiliations:** ^1^Florida Maxima Corporation, Orlando, FL, United States; ^2^Air Force Research Laboratory, Dayton, OH, United States

**Keywords:** fluid teams, teams, composition, team performance, team composition

## Abstract

The need exists to better understand how to comprise fluid teams—teams that are assembled on short notice, from members with little to no familiarity, who come together to carry out a time-limited task, and then disband. Due to the ever-increasing complexity of the modern workplace, the demand for these types of fluid teams is growing in task domains such as the military, aviation, healthcare, and industry. The aim of this paper is to review the team composition literature to shed light on composition considerations for forming fluid teams.

## Introduction

Thanks to more nuanced models of team composition, novel research and analytic methods, and a plethora of meta-analytic integrations, we know a good deal about what makes teams tick. While there is still much to be learned, the question of how to best compose a team has become clearer. A cumulative body of research points to various attributes that, when combined, promote or detract from team processes and performance. Given that the weight of empirical evidence is based on traditional teams, the question remains to what degree does the current state of the science on team composition generalize to fluid teams. Consequently, the aim of this paper is to shed light on this issue. To accomplish this, we first address the concept of fluid teams and consider how they differ from traditional teams. Second, we review the team composition literature with a focus on fluid teams and summarizing the current state of the science on fluid team composition.

## Fluid teams

Research on teams has traditionally focused on the study of team inputs, processes, emergent states, and outputs of intact teams, or teams with a relatively stable membership. Given this focus, much is now known about the workings of these types of teams (Driskell et al., 2018). However, research over the years has also examined the operation of *ad hoc* or temporary teams. Ziller (1965), in a paper titled *Toward a theory of open and closed groups* differentiated between open vs. closed groups. Open groups were characterized as having unstable membership (team members may be added, subtracted, or replaced in the group), reduced time perspective (tending to be short-lived), and a continuous state of disequilibrium or instability. Other research has described similar team contexts in terms of *ad hoc* teams (Lorge et al., 1958), temporary teams (Saunders and Ahuja, 2006; Blomqvist and Cook, 2018), swift starting action teams (McKinney et al., 2005; Wildman et al., 2012), and emergent response teams (Majchrzak et al., 2007). Driskell et al. (2023) provide a specific definition of a *fluid team* as comprised of four core characteristics; (1) team members are rapidly assembled to address an immediate problem, (2) members are assembled based on domain expertise and typically have no prior history or experience working together, (3) the team must begin work on a task that is immediate, time-critical, and of short duration, and (4) at completion of the task, the team disbands with little likelihood of further interaction. These types of teams, assembled temporarily from experts across various domains to accomplish critical, time-sensitive tasks, have become increasingly prevalent in various contexts such as the military, aviation, healthcare, and industry (Bell et al., 2023, this issue; Grossman et al., 2024, this issue; Linhardt and Salas, 2023, this issue; Capiola et al., 2020). Although fluid teams are typically convened to address complex and demanding tasks, little is known regarding how these teams should be assembled to support effective performance in this unique context.

## Team composition

Team composition research is concerned with identifying and arranging the sets of attributes present among team members that facilitate effective team performance. The combination of team member attributes serves to moderate team performance through impacting the attitudes, behaviors, and cognitions of teams during task performance (Bell et al., 2018). Some of the more well-studied attributes that team composition research has examined include demographic diversity (e.g., Carter et al., 2019), task-related diversity (e.g., Joshi and Roh, 2009), cognitive ability (e.g., Devine and Phillips, 2001), personality (e.g., Bell, 2007; Driskell and Salas, 2013), and collective orientation (Driskell et al., 2010).

When examining individual level attributes—that is, what individual team members bring to the team—composition researchers are tasked with two primary objectives: (1) identifying the type of attributes that contribute to effective team performance, and (2) determining how these attributes should be configured within the team. Stewart (2006) noted that “One line of research examines aggregated characteristics to assess whether the inclusion of individuals with desirable dispositions and abilities improves team performance. A related but somewhat different area of research looks at how heterogeneity of individual characteristics relates to team outcomes” (p. 30). While related, these aggregation approaches represent a useful categorization method when trying to explain the relationship between selection criteria and team outcomes.

Given that fluid teams represent an understudied type of team, the collective wisdom on how to compose fluid teams is limited. The prevailing approach to assembling rapid forming fluid teams is to simply match expertise to the task with little to no thought about attributes outside of sheer capability. The following sections on team composition deal specifically with how the distribution of individual attributes among a team combine to influence team performance. That is, the extant research on group composition for both surface-level attributes and deep-level attributes will be reviewed and the relationship between these attributes and team performance identified.

## Analytic approach

**Figure 1:**
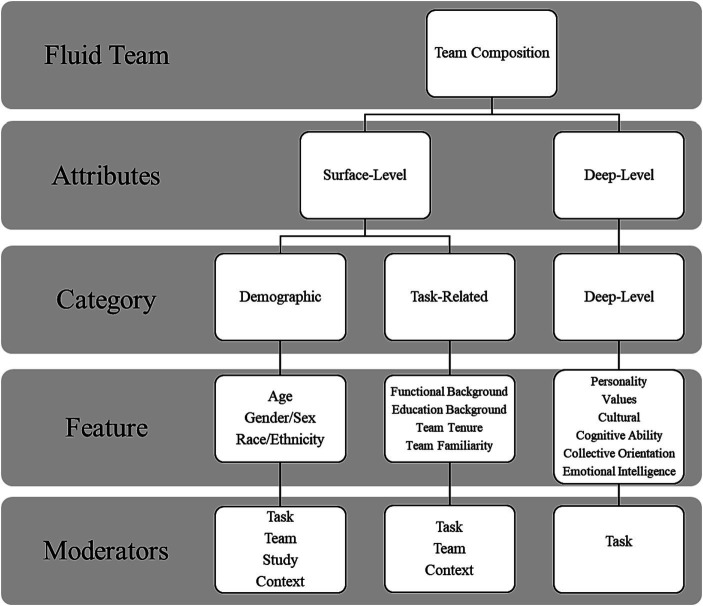
Overview of the review strategy.

Figure 1 serves as an advanced organizer for this review. Because this line of research is necessarily complex, a guiding framework will assist the reader in the following exposition. For the purposes of this review, we dichotomize team member attributes as surface-level or deep-level attributes. Surface-level attributes of team members refer to overt easily distinguishable cues such as age or gender that are readily perceptible in short-term interactions. Composition researchers have further classified surface-level attributes as demographic attributes (e.g., gender, race, and age) or task-related attributes (e.g., educational level, organizational position). This dichotomization maps to the two predominant perspectives explaining the diversity-performance relationship: The social categorization/intergroup bias/similarity–attraction perspective (e.g., Byrne, 1971; Turner, 1985) and the informational diversity–cognitive resource perspective (e.g., Cox and Blake, 1991). The social categorization/intergroup bias/similarity–attraction perspective proposes that homogeneous teams (whose team members are similar in demographic characteristics such as gender, age, socioeconomic status) should outperform diverse teams due to mutual attraction among team members and social categorization processes, which can foster cooperation. The informational diversity–cognitive resource perspective proposes that diversity (especially in terms of task-related attributes such as expertise or functional background) should provide a greater corpus of knowledge, skills, abilities, and perspectives to draw upon, which could enhance team performance. As Figure 1 illustrates, we intend to examine both demographic attributes and task-related attributes that have been examined in the team composition literature, as well as relevant moderators of these effects.

We also examine deep-level attributes such as values or personality that tend to manifest themselves over time (Driskell et al., 2006; Bell, 2007). Broadly, we expect surface-level attributes to be more strongly predictive of team performance in fluid teams. This assertion is based on the notion that information regarding deep-level attributes (e.g., personality, values, and beliefs) cannot be easily conveyed in a fluid team context in which the team members are rapidly assembled, with little or no familiarity, and perform a short-term, time-limited task. As Harrison et al. (2003, p. 639) note, fluid team members have “neither initial familiarity nor a chance to develop familiarity over time.” Thus, a longer team tenure allows information to be conveyed between team members, which in turn, allows team members to unmask deep-level attributes via self-disclosure. More recent research on faultlines has also supported this claim (Zhang and Chen, 2023). However, deep-level attributes are still expected to impact performance in fluid teams, especially in instances in which opportunities exist to engage in mutual self-disclosure, when value differences are foreknown, or when surface-level attributes such as organizational affiliation may imply value differences. Moreover, some deep-level attributes such as conscientiousness may be easily construed in a task environment where proxy cues such as hard-working behavior are readily available.

The review process included searching online databases (e.g., PsychInfo) for relevant literature using the search terms *team composition* and *team performance*, and then using an ancestry search approach to locate relevant studies from the reference lists of obtained studies. In addition, the review and analysis rely heavily on a set of meta-analytic integrations that have been conducted on team composition (e.g., Bell, 2007; Joshi and Roh, 2009; Bell et al., 2011; van Dijk et al., 2012; Carter et al., 2019; Gonzalez-Mulé et al., 2020; Niler et al., 2021; Triana et al., 2021; Byron et al., 2023; Kilcullen et al., 2023) (Summary data from these meta-analyses are provided in a Supplementary Table 1) Most of the relevant obtained studies have been published in the last two decades and published in a wide range of journals, including the Journal of Applied Psychology, Journal of Management, Personnel Psychology, Small Group Research, and Journal of Organizational Behavior, among others.

Furthermore, given that the results of these prior analyses identify a large number of predictor-performance relationships and moderators, we try to focus our discussion on those studies and results most potentially relevant to fluid teams. For example, recent research has examined how faultlines can change and evolve over time (Meister et al., 2020) but this is less relevant to fluid teams that operate and then disband within a short time frame. In many cases we seek to “read between the lines” to identify certain proxies that can be leveraged to glean information about fluid team composition. One such proxy that has been examined in the various meta-analytic integrations is study setting (i.e., laboratory studies versus field studies). As Bell et al. (2011, p. 13) note, “Study setting is…likely to be highly correlated with the length of time that a team has been together.” That is, laboratory studies are likely to be composed of *ad hoc* teams compared to field studies, which often are comprised of intact teams. Thus, this comparison may shed light on fluid team composition. That said, caution should be taken when trying to draw direct comparisons.

## Surface-level attributes

### Surface-level diversity: demographic

Demographic attributes represent fairly immutable characteristics of individuals such as age and sex. The following sections describe the overall composite demographic diversity-performance relationship, followed by the diversity-performance relationship for the most studied individual attributes.

Overall, composite demographic diversity (i.e., demographic diversity considered as a unidimensional or composite variable) has been shown to have a weak negative to near zero relationship with performance (see Webber and Donahue, 2001; Stewart, 2006; Horwitz and Horwitz, 2007; Joshi and Roh, 2009; van Dijk et al., 2012). However, looking only at the overall mean effect size of the relationship between a specific variable and the relevant outcome measure can mask important differences, such as how the effect size increases or decreases as a function of some theoretically relevant and practically important moderators. The most relevant moderating variables are outlined below.

#### Task characteristics

##### Task difficulty/complexity

Shuffler and Carter (2018) define task complexity as a context that is ambiguous, multifaceted, dynamic, and demands a sense of urgency. Meta-analyses examining the moderating effects of task difficulty/complexity suggest that demographic diversity may adversely impact teams performing simple tasks (i.e., effect sizes range from small negative to near zero) and have little effect on performance for more difficult tasks. Bowers et al. (2000) found that homogenous teams performed moderately better on low difficulty tasks and heterogenous teams performed better on high difficulty tasks. van Dijk et al. (2012) also examined the moderating effects of task complexity but did not find moderation for task complexity. Lastly, Wang et al. (2019) found that surface-level diversity was detrimental to performance, operationalized as creativity/innovation, on simple tasks, but unrelated to performance for complex tasks. Thus, to the extent that fluid teams are primarily convened to address complex, critical tasks, this suggests that negative effects of demographic diversity on fluid team performance may be weak.

##### Task interdependence

Demographic diversity can lead to process loss (e.g., via increased conflict) and such disruptions during task performance have the potential to be detrimental. This would particularly be the case when interdependence is high. That is, interdependent tasks rely on efficient intra-team communications and disruptions to this should adversely impact performance. Joshi and Roh (2009) found that for low interdependence tasks there was a small positive relationship to performance, moderate levels of interdependence showed a small negative relationship, and high interdependence showed a small to near zero relationship to performance. As Joshi and Roh note, these results suggest “that the interactive effects of team diversity and interdependence may be more complex than acknowledged in the past” (p. 620).

##### Team cognition

Team cognition refers to emergent states that determine how task and team knowledge is organized among team members (e.g., transactive memory, team mental models). Although team cognition has been shown to be a robust predictor of team performance, Niler et al. (2021) examined moderators of this overall effect. They proposed that team homogeneity would weaken the relationship between team cognition and team performance, and results indicated that the team cognition-performance relationship was significantly stronger for heterogeneous teams (varying in demographic attributes such as race, gender, or age) than homogenous teams. This moderation was not significant for teams with varying functional diversity (e.g., expertise, functional background). This suggests that team cognition is most strongly related to team performance when the team members are more demographically diverse. The authors note that demographic diversity can trigger social categorization processes that can negatively impact information processing, rendering team cognition processes more important in supporting performance.

#### Team characteristics

##### Team tenure

Team tenure, or the longevity of team membership, is of direct relevance to fluid teams. Examining short-term vs. long-term teams—a proxy for tenure or longevity, Joshi and Roh (2009) found that relations-oriented diversity (similar to demographic diversity) demonstrated a positive relationship to performance in short-term teams and a negative relationship to performance in long-term teams (defined as teams working together for longer than 2 years). Joshi and Roh suggest that the urgency surrounding short-term team task performance may lead team members to overlook differences and instead require them to leverage these differences during task performance. Moreover, these authors suggest that in long-term teams, “divisions based on diversity attributes may become more entrenched and self-reinforcing, so that conflicts and differences based on relations-oriented attributes have a more debilitating impact on team performance” (p. 611). Although these results imply that demographic diversity would have a stronger effect in long-term teams versus short-term teams, the operationalization of short versus long-term teams as either less or more than 2 years duration may yield unique results compared to other operationalizations of team tenure. The authors note that more research is needed to better understand the moderating relationship of team tenure. For example, Byron et al. (2023) found team tenure to be curvilinearly related to team outcomes of innovation and creativity, thus suggesting that this relationship may be more complex than previously thought.

#### Study characteristics

##### Diversity conceptualization

How diversity is conceptualized has an impact on the diversity-performance relationship. Harrison and Klein (2007) outlined three types of diversity including variety, disparity, and separation. These types of diversity relate to different theories and consequently predict varying outcomes. *Variety* represents a uniform distribution of an attribute among a team. Minimum variety would occur if every member had the same level or characteristic of that attribute. Maximum variety would occur when every individual has different levels of that attribute. Variety is related to the informational diversity–cognitive resource perspective and is predicted to result in greater creativity/innovation, better decision quality, more task conflict, and greater unit flexibility. *Disparity* represents an inequitable, positively skewed distribution of an attribute among a team such that one member is at the high end and the other members at the low end. Minimum disparity would occur if all members are equal on an attribute and maximum disparity occurs when one member is high and the others low. Disparity relates to status hierarchy and distributive justice (Harrison and Klein, 2007) and is predicted to result in more within-unit competition, reduced member input, withdrawal, and resentful deviance. Lastly, *separation* represents a bimodal distribution on an attribute such that half the team members are high and half the members low on an attribute. Minimum separation would occur if every member was the same on an attribute and maximum separation would occur when half were low and half high (or half one category and half the other; e.g., gender). Separation is related to the social categorization/intergroup bias/similarity–attraction perspective and is predicted to result in reduced cohesion, more interpersonal conflict and distrust, and decreased task performance.

Wei et al. (2021) investigated the moderating effect of diversity type on the relation-oriented (demographic) diversity-innovation relationship. Their findings demonstrate that relation-oriented variety had a negative, yet non-significant impact on corporate innovation. Relation-oriented disparity did not show a relationship with innovation. And relation-oriented faultline strength (i.e., equivalent to separation) showed a negative relationship with innovation.

##### Study setting

Examining studies that were carried out in laboratory or field settings, Bell et al. (2011) found that the harmful effects of demographic diversity were only demonstrated in field settings. Given that laboratory studies may be a broad proxy for *ad hoc* fluid teams, further research is needed to examine this relationship.

#### Contextual characteristics

##### Industry setting

Two meta-analyses examined the moderating effect of industry setting. Joshi and Roh (2009) found that diversity has the most detrimental impact on teams from high-technology industries, a small negative relationship for manufacturing teams, and a beneficial effect for service industry teams (see also Carter et al., 2019). To the extent that high-technology settings may represent more complex and interdependent task contexts, these results may also apply to fluid teams.

Overall, the findings from this literature suggest that demographic diversity can lead to process-loss in teams via increased levels of conflict, reduction in cohesion, and impaired information processing (e.g., Stahl et al., 2010). For example, Mesmer-Magnus and DeChurch (2009) found that information sharing (operationalized as uniqueness and openness) was greater for teams with greater surface-level similarity. However, whether an apparent process-loss impacts team performance depends on a variety of variables.

#### Individual attributes: age, gender, and race

Shifting from the examination of composite diversity measurement to more specific individual demographic variables, researchers have examined age, gender, and racial diversity.

Age diversity reflects chronological age differences between team members. Meta-analytic results show weak to near zero relationships between age diversity and performance [Bell et al., 2011 (*ρ* = −0.03); Byron et al., 2023 (*ρ* = 0.01); Carter et al., 2019 (*r* = 0.04); Joshi and Roh, 2009 (*r* = −0.06); van Dijk et al., 2012 (*r* = −0.03)]. Despite these insignificant findings, moderator analyses have shown significant variability among various moderating variables. For example, Carter et al. (2019) found that the age diversity benefits service industry teams and disadvantages high-technology teams.

Examining gender diversity, meta-analytic results show a small to near zero negative relationship between gender diversity and performance [Bell et al., 2011 [−0.06]; Bowers et al., 2000 (*z* = −0.38, *p* > 0.05); Byron et al., 2023 (*ρ* = −0.04); Carter et al., 2019 (*r* = −0.03); Joshi and Roh, 2009 (*r* = −0.02); Schneid et al., 2015 (*r* = −0.01); van Dijk et al., 2012 (*r* = −0.01)]. Moderator analyses have identified significant moderating variables. For instance, Bowers et al. (2000) found that gender homogenous teams performed better on simple tasks, while gender heterogenous teams performed better on difficult tasks. Schneid et al. (2015) examined the moderating effect of societal gender egalitarianism and found that gender diversity adversely impacts performance in low gender egalitarianism societies that do not promote gender equality (see also Feitosa et al., 2023). Lastly, examining the moderating effect of study setting, Bell et al. (2011) found sex diversity to be unrelated to performance in laboratory settings, yet negatively related to performance in field settings.

The race/ethnicity diversity-performance relationship has been shown to be negative to near zero [Bell et al., 2011 (*ρ* = −0.11); Byron et al., 2023 (*ρ* = 0.03); Carter et al., 2019 (*r* = −0.06); Joshi and Roh, 2009 (*r* = −0.01); van Dijk et al., 2012 (*r* = −0.05)], however, again, moderating factors are informative. Joshi and Roh (2009), examining occupational demography, found that race/ethnicity diversity in majority white occupations negatively impacts performance, while diversity promotes performance in balanced occupations. Stahl et al. (2010) investigated the relationship between cultural diversity and team processes, finding a positive relationship between cultural diversity (e.g., race/ethnicity) and conflict and a negative relationship between cultural diversity and communication effectiveness and social integration (e.g., cohesion). Bell et al. (2011) found that race diversity was more damaging to performance in field settings as opposed to laboratory settings. As mentioned previously, study setting may be viewed as a proxy for fluid teams. As Bell et al. note, this would lead one to predict a weaker relationship between demographic diversity and performance in field settings (i.e., they have been together longer and thus surface-level diversity should give way to deep-level diversity) and a stronger relationship in *ad hoc* teams, although further research is needed to apply these findings to fluid teams.

Future research may look beyond single attributes and examine the interaction of multiple attributes. That is, age alone, or any demographic attribute, is likely to be insufficient to explain the diversity-process-performance relationship. Research on faultlines—hypothetical dividing lines that may separate team members based on one or more attributes—has made progress in investigating the impact of multiple attributes on performance. Leveraging theories of social categorization, social identity and the similarity–attraction perspective, stronger faultlines (i.e., the alignment of multiple attributes) are predicted to have a greater impact on team process and performance. Several meta-analytic integrations have been able to examine this proposition. Results from meta-analytic integrations show a negative effect of demographic faultlines on performance [Carter et al., 2019 (*r* = −0.05); van Dijk et al., 2012 (*r* = −0.06); Wei et al., 2021 (*ρ* = −0.17)]. Moreover, Zhang and Chen (2023) found that surface-level social faultlines activate subgroup formation, which in turn adversely impacts team interaction quality and team performance.

### Surface-level diversity: task-related

Task or job-related attributes reflect team member characteristics expected to relate more directly to task performance (e.g., functional background). Leveraging the informational diversity–cognitive resource perspective (e.g., Cox and Blake, 1991), task-related diversity is expected to enhance performance in that task-related diversity should provide a greater corpus of KSAs to draw upon. In instances where a diverse range of expertise is needed for task performance, a diverse functional background should benefit performance. More specifically, diverse functional backgrounds should benefit performance when (a) a task requires varied functional backgrounds, (b) the varied functional backgrounds on the team are task-relevant, and (c) when teams have the resources and abilities to draw upon these backgrounds. Overall, task-related diversity has been shown to have a small positive relationship with performance (see Webber and Donahue, 2001; Horwitz and Horwitz, 2007; Joshi and Roh, 2009; van Dijk et al., 2012; Bui et al., 2019). Again, the overall findings have called attention to the importance of the following moderating variables.

#### Task characteristics

##### Task difficulty/complexity

van Dijk et al. (2012) was the only meta-analysis to directly examine task complexity as a moderating variable. Their findings show that task-related diversity is most beneficial when teams are executing complex tasks, in comparison to low or medium complexity tasks.

##### Interdependence

Joshi and Roh (2009) found some evidence for the beneficial effects of task-oriented diversity under moderate and high levels of task interdependence, as opposed to low interdependence.

#### Team characteristics

##### Team type (short vs. long-term)

Joshi and Roh (2009) found that the relationship between task-related diversity and performance was positive, yet very small to near zero, for both short and long-term teams.

#### Study characteristics

##### Diversity conceptualization

A single meta-analysis examined the moderating effect of diversity operationalization (e.g., variety, disparity, and faultline strength) on the general task-related-performance relationship (Wei et al., 2021). These authors found that each diversity operationalization was positively related to corporate innovation. Examining faultline strength, Zhang and Chen (2023) found surface-level task faultlines to benefit team interaction quality.

#### Contextual characteristics

##### Industry setting

Joshi and Roh (2009) found that task-related diversity was positively related to performance for high-technology teams and unrelated to performance for service industry and manufacturing teams.

##### Functional background

Functional background diversity refers to the “distribution of work history across the different functional specializations that exist within an organization” (Bell et al., 2011, p. 8). Meta-analytic results show a small positive relationship between functional background diversity and performance [Bell et al., 2011 (*ρ* = 0.10); Byron et al., 2023 (*ρ* = 0.06); Carter et al., 2019 (*r* = 0.06); Joshi and Roh, 2009 (*r* = 0.13); van Dijk et al., 2012 (*r* = 0.07)]. Moreover, moderator analyses have shown significant variability among variables. Examining team type, Bell et al. (2011) found functional background diversity to be most beneficial to design/cross-functional teams. Carter et al. (2019) found that similar functional backgrounds were most beneficial to manufacturing teams, whereas high-technology teams benefited the most from functional background diversity.

##### Educational background

Educational background refers to the content area of one’s education. The various meta-analyses examining educational background have shown no direct relation to performance [Bell et al., 2011 (*ρ* = 0.01); van Dijk et al., 2012 (*r* = 0.00)]. However, when the outcome of interest is creativity or innovation, a small positive effect is demonstrated [Byron et al., 2023 (*ρ* = 0.09); Bell et al., 2011 (*ρ* = 0.23)]. One explanation for the null results is that information about individual educational background is not often shared between team members. If these differences are not readily known by team members, as is fluid teams, the potential process gains from diverse educational backgrounds may not be realized.

##### Team tenure

Team tenure reflects how long team members have been on a team. Both Bell et al. and Carter et al. showed small, positive effects of team tenure on performance (*ρ* = 0.09, *r* = 0.09, respectively).

Team tenure diversity reflects the distribution of tenure among team members. Meta-analytic findings suggest the team tenure diversity-performance relationship is at or near zero [Bell et al., 2011 (*ρ* = −0.04); Byron et al., 2023 (*ρ* = 0.06); Carter et al., 2019 (*r* = −0.00); van Dijk et al., 2012 (*r* = −0.02)]. Thus, the variability of tenure among team members does not seem to impact performance to a significant degree.

A recent meta-analysis examining the team tenure-process-performance relationship deserves special attention (Gonzalez-Mulé et al., 2020). Gonzalez-Mulé et al. (2020) examined the relationship between (a) three types of tenure (i.e., additive, collective, and dispersion), (b) team processes (i.e., cognition, motivational-affective states, and behavioral processes), and (c) performance. Additive team tenure was defined as the average amount of time team members have spent in a given job, organizational role, or team. Collective team tenure was defined as the amount of time team members have spent together as members of the same team (e.g., time since last joining of a new member). And dispersion team tenure was defined as the variance of time in a job, organizational role, or team, equivalent to Harrison and Klein’s (2007) *variety* diversity. Gonzalez-Mulé et al. found that additive team tenure was positively related to performance, and that this relationship was mediated by team cognition. Collective team tenure was positively related to performance, and this relationship was mediated by motivational-affective states. Team tenure dispersion was also positively related to performance, and this relationship was mediated by team behavioral processes. Overall, they found additive team tenure to be most strongly related to team performance, followed by collective tenure, and then tenure dispersion. Moderator analyses found that the additive tenure-performance relationship was moderated by task interdependence such that additive team tenure is more strongly related to performance for highly interdependent tasks relative to less interdependent tasks, reflecting the importance of team cognition for interdependent tasks.

##### Team member familiarity

Carter et al. (2019) investigated whether the level of familiarity among team members relates to performance. While related to team tenure, familiarity captures instances whereby, for example, a newly added team member is known by the rest, or some, of the incumbent members. In instances where familiarity includes knowledge about work style, or member KSAs, familiarity can aid in, for example, team cognition (Bedwell, 2019). Results from Carter et al. showed that, overall, aggregated familiarity was not related to performance. Further research is warranted.

## Deep-level attributes

Deep-level attributes represent more deep-seated, difficult to discern characteristics about a person such as personality, values, attitudes, preferences, and beliefs (Harrison et al., 2002). Compared to surface-level attributes, deep-level attributes have been suggested to be more directly related to process and can have a stronger impact on performance (Bell, 2007; Triana et al., 2021), although, as we previously noted, deep-level attributes may not have the time to fully express themselves in short-term fluid teams. For deep-level attributes, higher average levels of desirable attributes among team members are generally viewed as desirable. For example, higher team average cognitive ability would be expected to be positively correlated with team performance. Thus, Stewart (2006) found that higher-levels of deep-level attributes (i.e., personality, cognitive ability, expertise) benefit team performance (*r* = 0.20). Diversity, or differing levels of deep-level attributes would reflect members with high and low scores on various attributes. The following sections review the overall composite deep-level diversity-performance relationship (including composite deep-level diversity, personality, values, and culture), followed by examination of the diversity-performance relationship for the most studied individual attributes.

### Deep-level diversity

The overall deep-level diversity-performance relationship has shown to be insignificant [Stewart, 2006 (*r* = −0.04); Triana et al., 2021 (*r* = −0.01); van Dijk et al., 2012 (*r* = −0.01)]. However, Triana et al. (2021) examined the impact of specific types of deep-level diversity (i.e., personality diversity, cultural diversity, and values diversity) on team processes and found that deep-level diversity showed a significant negative relationship with positive team emergent states, a significant negative relationship with team process, and a significant positive relationship with team conflict. Moreover, mediation analysis showed that the relationship between deep-level diversity and team performance was mediated by positive emergent states, positive team processes, and team conflict. Regarding faultline strength, Zhang and Chen (2023) found deep-level faultlines to be detrimental to team performance. Moreover, this research showed that this effect was serially meditated by subgroup formation and team interaction quality, respectively.

#### Task difficulty/complexity

van Dijk et al.’s (2012) meta-analysis examining the moderating effects of task difficulty/complexity suggests that deep-level diversity is detrimental to team performance in more complex tasks. This overall finding is in line with Triana et al. (2021) who found deep-level diversity to be more detrimental to team positive emergent states and team processes in tasks of high complexity. This suggests that on one hand, deep-level diversity may be detrimental to performance in complex fluid team tasks, but on the other hand, we have argued that deep-level attributes may be less impactful in such short-term time-compressed team contexts. Further research is needed.

### Composite personality diversity

Considering composite personality (aggregate scores of the Big Five personality traits), the overall personality diversity-performance relationship has been shown to be insignificant [Bowers et al., 2000 (ns); van Dijk et al., 2012 (*r* = 0.04)]. A recent meta-analysis by Triana et al. (2021) found that personality diversity exhibited a significant negative relationship with positive emergent states.

### Composite values diversity

Values diversity was examined in two meta-analyses. The direct values diversity-performance relationship was examined by van Dijk et al. (2012) and did not show a significant relationship between values diversity and performance (*r* = −0.07). More interestingly, Triana et al. (2021) found that values diversity showed a significant negative relationship with positive emergent states, a significant negative relationship with positive team process, and a significant positive relationship with team conflict. Thus, while more research is needed to examine the impact of values diversity on performance, values diversity seems to disrupt important team processes.

### Composite cultural diversity

Examining team processes, Triana et al. (2021) found cultural diversity to be unrelated to positive emergent states, unrelated to positive team processes, and positively related to team conflict (*ρ* = 0.24). Stahl et al. (2010) reported that overall cultural diversity was positively related to both task and relationship conflict, but unrelated to process conflict. Although all types of conflict have been shown to negatively impact team process and performance, this relationship has been shown to be complex (De Dreu and Weingart, 2003; de Wit et al., 2012; O'Neill et al., 2013). That is, some types of conflict may benefit certain teams in certain circumstances (e.g., creative problem-solving tasks). This relationship, however, has been shown in past research to be curvilinear, such that medium levels of task conflict have been shown to increase team innovation, while low and high levels can be detrimental (De Dreu, 2006). Potential positive effects of conflict aside, conflict should be viewed as a negative process. The research examining the conflict-trust relationship shows that all types of conflict degrade trust, and significantly so (relationship conflict, *ρ* = −0.45; task conflict, *ρ* = −0.53; process conflict *ρ* = −0.59; de Wit et al., 2012). Moreover, research suggests that the possible positive effects of conflict (specifically task conflict) may only be present when high levels of trust are already present among teams (De Dreu and Weingart, 2003). Balliet and Van Lange (2013) concur, concluding that “Trust matters most in situations that contain greater amounts of conflicting interests” (p. 1102). Cultural diversity may be particularly detrimental to fluid teams, given that initial levels of trust among unfamiliar team members may be low (Driskell et al., 2023).

### Personality

The personality-team performance relationship has focused on the distribution of the big-five personality traits (i.e., conscientiousness, agreeableness, extraversion, emotional stability, and openness to experience) among team members. The overall findings from meta-analyses that examined individual personality traits, as opposed to those collapsing personality traits into a single variable, have found elevated average trait levels to be either positively related or unrelated to performance and for variability in trait levels to be either negatively related or unrelated to performance (Peeters et al., 2006; Bell, 2007; van Dijk et al., 2012; Carter et al., 2019). These relationships, as well as significant moderators, are described in more detail below.

#### Conscientiousness

Conscientiousness has been described by Costa and McCrae (1992) as a dimension that contrasts organized, scrupulous, diligent people against lax, unorganized, and lackadaisical people. In team task settings, high mean levels of conscientiousness are desirable, whereas high variability or varying levels of conscientiousness may impede performance due to (a) tension, or conflict, arising from personality conflicts on this dimension, (b) from lackluster performance from low conscientious members, and/or (c) the requirement of other members to make up for potential process-loss attributed to low conscientious members.

In separate meta-analyses, Bell (2007) found average conscientiousness to be positively related to performance (*ρ* = 0.14), Peeters et al. (2006) found a positive relationship between conscientiousness and performance (*ρ* = 0.20), and Carter et al. (2019) also found aggregated conscientiousness to relate positively to performance (*r* = 0.08). Bell further reported that this relationship was only present in field studies as opposed to laboratory studies, whereas Peeters et al. (2006) found this relationship to be notably larger for professional teams versus student teams.

Meta-analytic efforts show the relationship between conscientiousness diversity and performance to be negative, yet effect sizes range from small to near-zero [Bell, 2007 (*ρ* = −0.03); Carter et al., 2019 (*r* = −12); Peeters et al., 2006 (*ρ* = −0.24); van Dijk et al., 2012 (*r* = −0.09)]. Bell further found detrimental effects of conscientiousness diversity only for field studies.

Taken as a whole, the findings suggest that team performance benefits from greater levels of overall conscientiousness and suffers from conscientiousness diversity. Although current data do not directly address this, these effects may be somewhat weaker in *ad hoc* fluid teams that operate over short time periods.

#### Agreeableness

Agreeableness has been described as a dimension of interpersonal behavior that contrasts persons who are trusting, sympathetic, and cooperative against persons who are callous, cynical and antagonistic (Costa and McCrae, 1992). Similar to conscientiousness, agreeableness is deemed to be a desirable trait to have among team members. Overall, aggregated levels of agreeableness are shown to be positively related to performance [Bell, 2007 (*ρ* = 0.12); Carter et al., 2019 (*r* = 0.14); Peeters et al., 2006 (*ρ* = 0.24)]. In regard to moderators, Bell found that agreeableness was most strongly related to performance when aggregation was operationalized as average (i.e., mean team agreeableness) and when operationalized as team minimum agreeableness (i.e., the low team member on agreeableness) suggesting that one disagreeable team member can impact the entire team. Similar to their findings regarding conscientiousness, the agreeableness-performance relationship was found to be only significant in field settings. Paralleling Bell’s findings, Peeters et al. showed elevated agreeableness to be only related to performance in professional teams.

Meta-analytic integrations examining the agreeableness diversity-performance relationship show a negative to near zero association [Bell, 2007 (*ρ* = −0.04); Carter et al., 2019 (*r* = −0.14); Peeters et al., 2006 (*ρ* = −0.12); van Dijk et al., 2012 (*r* = −0.03)]. Moderator analyses from these studies are non-significant.

The overall findings suggest that teams benefit from members who score high on agreeableness. Moreover, the results also highlight the importance of the member with the minimum level of agreeableness, suggesting an antagonistic, uncooperative individual can degrade the team. Variability on agreeableness seems to have a weak negative relationship with performance. Results may be weaker for *ad hoc* vs. field teams.

#### Extraversion

According to Costa and McCrae (1992), extraversion includes traits such as sociability, activity, and the tendency to experience positive emotions. It is worth noting that these lower-level traits that comprise the broader Extraversion factor may show differential effects. Some researchers differentiate between the sociability/affiliation sub-trait of Extraversion and the outgoing/assertiveness sub-trait (Driskell and Salas, 2013).

Concerning aggregated levels of extraversion, Bell (2007) found a small, yet positive relationship between extraversion and team performance (*ρ* = 0.10); Carter et al. (2019) replicated this finding (*r* = 0.10), but Peeters et al. (2006) found no relationship (*ρ* = 0.03). Bell’s findings show this relationship to only be significant for teams in field settings. Regarding variability, or diversity, no significant extraversion diversity-performance was found. The results of the meta-analyses reviewed suggest a small benefit to having team members with higher levels of extraversion, albeit perhaps weaker in *ad hoc* vs. field team settings. Moreover, diversity in extraversion has shown to be unrelated to team performance.

#### Emotional stability

Emotional stability reflects a person’s tendency to not experience psychological distress (Costa and McCrae, 1992). With lower emotional stability reflecting neuroticism, emotional stability would be deemed a desirable trait among team members. This may be particularly true when teams must perform under high demand or stress.

The meta-analyses examining aggregated levels of emotional stability generally support this claim. Bell (2007) showed a small significant relationship between average emotional stability and performance (*ρ* = 0.13), Carter et al. (2019) reported similar findings (*r* = 0.13), yet Peeters et al. (2006) found no significant relationship (*ρ* = 0.03). Of the three meta-analyses examining the aggregated emotional stability-performance relationship, the moderator analyses report mixed results, suggesting that the relationship between aggregated emotional stability and team performance is likely moderated by other variables (e.g., task difficulty, intrateam conflict, etc.). The findings regarding the emotional stability diversity-performance relationship demonstrate no significant relationship to performance.

Overall, the findings suggest a small performance gain for elevated aggregate levels of emotional stability on team performance. Variability in emotional stability does not seem to impact performance.

#### Openness to experience

Openness to experience refers to individuals who are imaginative and have a rich and complex emotional life, and who are intellectually curious, behaviorally flexible and nondogmatic in their attitudes and values (Costa and McCrae, 1992).

The findings regarding aggregated openness to experience are similar to those of extraversion and emotional stability. Bell (2007) and Carter et al. (2019) found a significant positive relationship between mean openness to experience and team performance (*ρ* = 0.11 and *r* = 0.13, respectively). When broken down by type of setting, Bell only found a significant positive relationship between openness to experience and performance for field studies. The meta-analytic integrations find the overall openness to experience diversity-performance relationship to be unrelated to team performance; although both Bell and Peeters et al. showed that diversity negatively impacted performance for teams in field settings.

Overall, the results suggest that teams benefit from individuals with greater openness to experience. Diversity may be detrimental to performance in field settings versus laboratory settings. As noted with other personality traits, openness to experience may not have opportunity to express itself in time-compressed, short-duration tasks.

### Cognitive ability

Cognitive ability, also referred to as general mental ability, reflects an individual’s ability to process information and learn. In regard to team performance, one would expect that teams with higher levels of cognitive ability would outperform teams of lower levels of cognitive ability. Meta-analyses support this relationship [Bell, 2007 (*ρ* = 0.30); Carter et al., 2019 (*r* = 0.07; ns); Devine and Phillips, 2001 (*r* = 0.29); Stewart, 2006 (*r* = 0.30)]. Both Devine and Phillips as well Bell examined study setting as a potential moderator. Devine and Phillips found the cognitive ability-performance relationship to be stronger in laboratory settings than in field settings, whereas Bell’s results did not show significant subgroup variation.

Diversity in cognitive ability would reflect team members with varying levels of cognitive ability. The meta-analyses examining the cognitive ability diversity-performance relationship have shown diversity to be unrelated to performance (Devine and Phillips, 2001; Bell, 2007; van Dijk et al., 2012; Carter et al., 2019). The overall findings suggest that selecting team members high on cognitive ability benefits team performance.

### Collective orientation

Several concepts, including collective orientation, team orientation, and preference for teamwork, collectively reflect an individual’s propensity to work in a collective manner in team settings (Driskell et al., 2010). A related concept, collectivism, originally viewed at the national-level (Hofstede, 1980), but since applied to the individual-level (Triandis, 1995), reflects an emphasis on the collective over the individual. Taken as a whole, a focus on the team over the self should benefit teamwork and team performance.

Bell’s (2007) meta-analysis showed a significant positive aggregated preference for teamwork (*ρ* = 0.23) and collectivism (*ρ* = 0.31) team performance relationship. In both instances, the relationship to performance was only significant for teams in field settings. A recent more focused meta-analysis by Kilcullen et al. (2023) showed a positive impact of team orientation on team process and performance. Specifically, they found team orientation to be positively related to communication, coordination, cooperation, trust, shared mental models, backup behaviors, cohesion, innovation, satisfaction, leadership, and team performance and negatively related to conflict. Collectivism and preference for teamwork operationalized as diversity was not shown to be related to performance (Bell, 2007). Consequently, the findings suggest, unsurprisingly, that teams benefit from individuals who have a predilection for teamwork.

### Emotional intelligence

Emotional intelligence (EI) is described as the capacity to perceive, use, regulate, and understand both one’s own emotions and the emotions of others (Joseph and Newman, 2010). Bell (2007) found average team emotional intelligence to be positively related to team performance (*ρ* = 0.18), and this relationship was stronger in lab settings than in field settings, somewhat paradoxically. Joseph and Newman (2010, p. 54) concluded that “EI positively predicts performance for high emotional labor jobs and negatively predicts performance for low emotional labor jobs.” Thus, the impact of EI on team performance may be dependent on the task environment; when task settings do not allow for much emotional expression, EI should have less impact.

## Implications for forming fluid teams

As previously noted, fluid teams are unique in that they are rapidly assembled to execute critical, time-limited tasks and are composed of members who typically have no prior familiarity or experience working together, and who disband upon task completion. The temporary nature of these teams leads to several important implications. First, given their short nature, surface-level attributes should have a greater impact on fluid teams than deep-level attributes. That said, deep-level attributes should still impact fluid team process and performance, especially when opportunities exist for team members to engage in mutual self-disclosure, when member attributes are foreknown (e.g., military values), and when deep-level attributes are discernable (e.g., conscientiousness). Second, it is conceivable, and some of the data reviewed suggests this as a possibility, that diversity may have a lesser impact on fluid teams. That is, for example, the requirement to “hit the ground running” may override adverse diversity effects by reducing the time to “categorize” teammates and increasing the motivation to execute near-term tasks. Moreover, the knowledge that team membership is temporary may lead members to overlook diversity (e.g., there is no need to be concerned about future interactions). Furthermore, members of fluid teams are expected to have had more experience working on multiple teams. Exposure to different types of team members may also lessen diversity effects. The diversity in knowledge and skills gained from working on multiple teams can be imported to newly formed groups conferring an advantage to fluid teams. Third, fluid teams are faced with the obstacles presented to all new teams with the added difficulty of carrying out critical, time-limited tasks often characterized by high levels of complexity. That is, new teams lack the “know-how” to be an effective team, whereas longer-term or intact teams develop this knowledge over time. Fluid teams are unlikely to have the time to develop these team knowledge structures and as such are at a significant disadvantage.

In addition to the implications noted above, we review the most important implications drawn from the review in Table 1.

**Table 1:** Summary implications: universal.

Implications	Main takeaway
*Composition bonus effects*	When composing fluid teams to address a near-term problem or task, selection of team members on specific surface-level and deep-level characteristics in addition to technical ability may yield incremental gains in team performance.
*Potential for process-loss*	Demographic diversity can lead to process-loss and negatively impact team performance. These adverse effects are expected to be amplified when multiple attributes align to form stronger faultlines.
*Benefit of diverse KSAs*	Task-related diversity has been shown to be positively related to team performance. These findings are most central for functional background diversity, for performance measured as creativity/innovation, and for complex/interdependent tasks.
*Benefit of elevated levels of deep-level attributes*	Teams benefit from elevated aggregate levels of positive deep-level attributes. Moreover, deep-level diversity has been shown to be detrimental to team positive emergent states and team processes. Deep-level diversity has also been shown to negatively impact performance, as well as team positive emergent states and team processes, on more difficult/complex tasks.
*Larger potential impact of surface-level attributes*	Surface-level attributes may have a strong impact on fluid team performance given that team members lack familiarity with one another and must form initial task expectations for others in the team from overt, readily accessible differentiating information. Correspondingly, deep-level attributes may have a somewhat weaker impact on fluid team performance given that these attributes are typically expressed over time as the team interacts, and fluid teams typically operate over a short duration and then disband.

In addition to these universal implications, we highlight some of the more relevant discrete implications drawn from the findings in Table 2.

**Table 2:** Summary implications: discrete.

Implications	Main takeaway
*Surface-level attributes*	
*Observable process decrements*	van Dijk et al. (2012) found almost no relationship between demographic diversity and objective performance. However, subjective observer ratings indicated that diversity adversely impacted performance. This suggests that perceptible decrements were observed by outside raters and thus implies that process issues were apparent (e.g., intrateam conflict), but did not translate into objective performance decrements. This points to an overall observation that process decrements may be detrimental to the team even if they do not meet a threshold of impacting objective performance. Process decrements may especially impact fluid teams performing complex, demanding, highly interdependent and time-limited tasks.
*Potential for opposing effects*	The two prominent perspectives leveraged to explain the diversity-performance relationship—the social categorization/intergroup bias/similarity–attraction perspective and the informational diversity–cognitive resource perspective—may impact performance in opposite directions. That is, for example, demographic diversity may adversely impact performance whereas task diversity may benefit performance. The primary implication is that a thorough understanding of a team’s composition, including how various attributes present among a team combine to impact performance is needed.
*Identify, understand, and theoretically map diversity effects*	How diversity is operationalized (i.e., variety, separation, disparity) may have an important impact on team outcomes. That is, how diversity is conceptualized (i.e., as variety, disparity, or separation; Harrison and Klein, 2007) has an impact on the diversity-performance relationship. These types of diversity relate to different theories and consequently predict varying outcomes.
*Importance of task-related diversity is task/context specific*	Diverse functional backgrounds should benefit performance when (a) a task requires varied functional backgrounds, (b) the varied functional backgrounds on the team are task-relevant, and (c) when teams have the resources and abilities to draw upon these backgrounds.
*Diversity can breed creativity*	Diversity seems to benefit creativity or innovation. Team tasks that require innovation (vs. production) may benefit from diversity.
*Demographic attributes such as age, gender, or race may structure initial team interaction*	Initial team interaction is often structured according to observable surface level cues such as gender, race, or occupational status, and these task expectations are then modified by subsequent team interaction. In fluid teams that only operate within a short time span, surface-level cues may be more impactful, and can lead to stereotype-based interaction.
*Some cues may be more impactful than others*	Gender and ethnicity are overt, easily accessible cues, whereas cues such as educational background or expertise may be less immediately discernable. Salient cues are more likely to be used by individuals to make attributions, and consequently, more likely to impact their behavior.
*Weak team cognitions may make fluid teams more vulnerable*	Fluid teams may suffer due to undeveloped team cognitions. Moreover, the potential requirement of fluid teams to effectively execute challenging interdependent tasks that benefit from having high levels of team cognition suggests that fluid teams may be more vulnerable to diversity-based disruptions.
*The importance of context*	A number of meta-analytic results found observed negative effects of demographic diversity to be stronger for complex tasks, interdependent tasks, and tasks in high technology settings. Compared to traditional teams, fluid teams are more likely to be assembled to address such complex, high-demand tasks. On the other hand, results generally found the harmful effects of demographic diversity to be stronger in field settings than in *ad hoc* laboratory settings. While this may be an artifact of laboratory settings (e.g., reduced motivation), it may be that short-term teams without a chance for future interaction may be less impacted by demographic diversity. Further research is needed.
*Deep-level attributes*	
*Presence of multiple concurrent effects*	Diversity of deep-level attributes, though also applicable to surface-level attributes, can impact process/performance in multiple ways. For example, varying levels of conscientiousness may impede performance due to (a) tension, or conflict, arising from personality conflicts on this dimension, (b) from lackluster performance from low conscientious members, and/or (c) the requirement of other members to make up for potential process-loss attributed to low conscientious members. These multitudinal effects require further consideration.
*Personality*	Findings suggest that team performance benefits from greater levels of overall conscientiousness and suffers from conscientiousness diversity. Teams benefit from members who score high on agreeableness, and even a single antagonistic, uncooperative individual can negatively impact the team. Variability on agreeableness has a weak negative relationship with performance. Research suggests a small benefit of having team members with higher levels of extraversion, which we would expect to be dependent on the type of task. The findings suggest a small performance gain for elevated aggregate levels of emotional stability on team performance. Results suggest that teams may benefit from individuals with more openness to experience. The overall findings of the effects of personality on team performance must be qualified when applied to fluid teams, as results are often shown to be weaker in *ad hoc* teams than in field team settings.
*Collective orientation matters*	Teams benefit from individuals who have a predilection for teamwork (i.e., collective orientation). Having a teamwork focus may be especially important for teams that are rapidly assembled to address a short-term critical task.
*Other individual-level factors may be relevant*	The overall findings suggest that selecting team members high on cognitive ability will benefit team performance. Given that fluid teams engage primarily in task-related activities with less emphasis on social activities, cognitive ability may be especially salient. Emotional intelligence is positively related to team performance and this effect is stronger in field versus *ad hoc* teams. Short-term *ad hoc* teams may not offer the opportunity for emotional intelligence to express itself.
*Deep-level diversity can impact team processes and emergent states*	Deep-level diversity, including personality diversity, values diversity, and cultural diversity can have a negative impact on positive emergent states, positive team processes, and a positive relationship with conflict. Triana et al. (2021) found values diversity to most adversely impact team processes and states, followed by personality diversity, and then cultural diversity.
*Diversity can lead to conflict that degrades trust*	Trust is expected to be central to newly formed teams. Research examining the conflict-trust relationship shows that all types of conflict degrade trust (relationship conflict, *ρ* = −0.45; task conflict, *ρ* = −0.53; process conflict *ρ* = −0.59; de Wit et al., 2012). Moreover, research suggests that the possible positive effects of conflict (specifically task conflict) may only be present when high levels of trust are already present among teams (De Dreu and Weingart, 2003). This may be especially relevant to fluid teams where initial trust development is difficult (Driskell et al., 2023).
*Task complexity moderates the impact of deep-level diversity*	Meta-analysis of van Dijk et al. (2012) examining the moderating effects of task difficulty/complexity suggests that deep-level diversity is detrimental to team performance in more complex tasks. This overall finding is in line with Triana et al. (2021) who found deep-level diversity to be more detrimental to team positive emergent states and team processes in tasks of high complexity.
*Deep-level attributes may not be readily discernable in short-term team contexts*	Examining deep-level composition variables, Bell (2007) found that personality attributes and preference for teamwork were more strongly related to performance in field vs. laboratory studies. While again, this may be an artifact of laboratory studies, this finding suggests that the effects of deep-level attributes may be less salient in a short-term fluid team context. However, some characteristics, such as very low conscientiousness or very low emotional stability, may be immediately impactful. Further research is needed.

## Research gaps

The relationships between team composition and process/performance are extremely complex, however this literature is robust and comprehensive. Although *ad hoc* or temporary teams have been of interest to researchers for some time, the type of fluid teams that we conceptualize are largely a product of modern demands, in that many task contexts require immediate attention to near-term problems for which domain experts must be drawn from various disciplines, departments, or organizations. To an extent, the utilization of fluid teams in modern contexts such as industry, healthcare, and the military has outstripped our understanding of how to best support fluid team performance. Therefore, the majority of the implications that we can derive from the broad literature on team composition must be elaborated and examined in a fluid team context. Most of this work has yet to be done. In a separate article in this issue, Driskell et al. (2024) provide an overview of the critical research gaps and opportunities to support selection, training, and workplace design for fluid teams.

## Conclusion

As Bell et al. (2018) note, some combinations of team members work better than others. This is perhaps even more the case for unique contexts in which certain teams may operate. This review examines the weight of cumulative evidence on team composition that can be leveraged to better understand the determinants of fluid team effectiveness. We hope this discussion provides a foundation for continuing research on this topic.

## Data availability statement

The original contributions presented in the study are included in the article/Supplementary material, further inquiries can be directed to the corresponding author.

## Author contributions

TD: Writing – original draft. GF: Writing – review & editing. MT: Writing – review & editing. AC: Writing – review & editing. JD: Writing – review & editing.
