# Supplementary material for: Composition considerations for fluid teams: a review
Source: Front Psychol. 2024 Feb 12;15:1302022. doi: 10.3389/fpsyg.2024.1302022 (PMC10894934; doi:10.3389/fpsyg.2024.1302022)
Supplement: Supplementary file 1 [file Data_Sheet_1.PDF]

Table 1. Surface-level Attributes: Demographic

**Attribute**	**Performance**	**Moderators**	**Reference**
Gender	Composition (*homogeneous=heterogeneous*) Fisher's Z = -0.38, p > .05	<u>*Overall diversity*</u><br>Task difficulty<br>*Low* (*homogeneous>heterogeneous*) Fisher's Z= 1.85, p < .05<br>*Medium* = ns<br>*High* (*homogeneous<heterogeneous*) Fisher's Z= –2.37, p < .01<br><br>Task Type<br>Intellectual tasks (*homogeneous=heterogeneous*) Fisher's Z= –2.21, p = 0.99<br>Performance tasks (*homogeneous>heterogeneous*) Fisher's Z = 3.30, p < .01	Bowers et al., 2000
Bio-demographic diversity	*Performance: Quality* ρ= -.006 [-.09, .08]<br>*Performance: Quantity* ρ= -.02 [-.35, .30]<br>*Social integration* ρ= -.02 [-.08, .04]	<u>*Bio-demographic diversity*</u><br>Task Complexity – ns<br>Team type – ns<br>Criterion report type – ns<br>Criterion measure type – ns<br>Study setting – ns	Horwitz & Horwitz, 2007
Relations-oriented diversity	*Relations-oriented diversity r = -.03 [-.05, -.02]*<br>*Gender r =-.02 [-.04, .01]*<br>*Occupational demography*<br>*Maj. male setting r = -.09 [-.12, -.05]*<br>*Balanced r = .11 [.06, .15]*<br>*Race/ethnicity r =-.01 [-.04, .01]*<br>*Occupational demography*<br>*Maj. white setting r = -.07 [-.10, -.04]*	<u>*Relations-oriented diversity*</u><br>Occupational demography (see left column)<br>Industry setting<br>*High-tech r = -.18 [-.20, -.15]*<br>*Service r = -.07 [.05, .09]*<br>*Manufacturing r = -.04 [-.07, -.01]*<br>Interdependence	Joshi & Roh, 2009

	Balanced r = .11 [.07, .14]<br>*Age r =-.06 [-.09, -.04]*<br>*Occupational demography*<br>*Maj. younger setting r = -.08 [-.10, -.05]*<br>*Balanced r = -.05 [-.10, -.00]*	*Low r = .08 [.06, .10]*<br>*Moderate r = -.12 [-.14, -.10]*<br>*High r = -.04 [-.11, .03]*<br>Team type (team duration)<br>*Short-term r = .09 [-.01, .16]*<br>*Long-term r = -.14 [.02, .07]*	
Social categorization diversity	***Performance***<br>*Overall* ρ= -.024<br>***Process***<br>*Open Com.* ρ= .13<br>*Freq. of Com.* ρ= -.007	<u>*Social categorization diversity*</u><br>Uncertainty<br>*Low* ρ= -.046 [-.17, .08]<br>*High* ρ= -.010<br>Frequency of communication<br>ρ= -.14	Bui et al., 2019
Surface-level similarity	***Process*** *[90%CI]*<br>*Overall information sharing* ρ= .22 [.10, .34]<br>*IS uniqueness* ρ= .27 [.14, .40]<br>*IS openness* ρ= .18 [.02, .34]		Mesmer-Magnus & DeChurch, 2009
Race	*Overall* ρ= -.11 [-.14, -.05]<br>*Lab* ρ= .02 [-.03, .08]<br>*-variety* ρ= .00 [-.06, .07]<br>*Field* ρ= -.13 [-.18, -.06]<br>*-variety* ρ= -.13 [-.18, -.06]<br>*-efficiency* ρ= -.04 [-.07, -.01]<br>*-general perf.* ρ= -.14 [-.20, -.05]<br>*-creativity/innovation* ρ= -.18 [-.34, .01]	Study setting (see left column)<br>Diversity operationalization (see left column)<br>Performance operationalization (see left column	Bell et al., 2011

Sex	*Overall* ρ= -.06 [-.09, -.02]<br>*Lab* ρ= .02 [-.06, .09]<br>*-variety* ρ=.07 [.00, .11]<br>*Field* ρ= -.07 [-.11, -.02]<br>*-separation* ρ= -.01 [-.17, .14]<br>*-variety* ρ= -.09 [-.12, -.04]<br>*-efficiency* ρ= -.09 [-.14, -.03]<br>*-general perf.* ρ= -.06 [-.11, .00]<br>*-creativity/innovation* ρ= -.16 [-.29, -.00]	Study setting (see left column)<br>Diversity operationalization (see left column)<br>Performance operationalization (see left column	Bell et al., 2011
Age	*Overall* ρ= -.03 [-.06, .01]<br>*Lab* ρ= .07 [-.06, .18]<br>*Field* ρ= -.03 [-.07, .01]<br>*-separation* ρ= .04 [-.10, .18]<br>*-variety* ρ= .01 [-.06, .07]<br>*-disparity* ρ= -.04 [-.08, .02]	Study setting (see left column)<br>Diversity operationalization (see left column)	Bell et al., 2011
Demographic diversity	<u>*Creativity/innovation*</u><br>*Overall demographic diversity* ρ= .01 [-.03, .05]<br>*Gender* ρ= -.04 [-.09, .01]<br>*Age* ρ= .01 [-.04, .06]<br>*Racial/ethnicity* ρ= .03 [-.08, .14]<br>*Educational level* ρ= .00 [-.07, .07]	<u>*Supplemental Analyses*</u><br>*Demographic diversity*<br>*Country culture*<br>*Collectivistic* ρ= *.05 [-.00, .09]*<br>*Individualistic* ρ= *-.09 [-.17, .00]*<br>*Team collaboration* ρ= -.06 [-14, .03]	Byron et al., 2022
Age	*Composition: Aggregated r= .04 [.01. .07]*<br>*High tech r= .05 [-.07, .18]*<br>*Manufacture r= .06 [-.02, .13]*	*Industry type (see left column)*	Carter et al., 2019

	Service r= .02 [-.03, .08]<br>*Student r= .04 [-.02, .11]*<br>*Composition: Heterogeneous r= -.03 [-.09, .03]*<br>*High tech r= -.22 [-.31, -.13]*<br>*Manufacture r= .06 [-.19, .31]*<br>*Service r= .12 [.05, .19]*<br>*Student r= -.02 [-.11, .06]*		
Race/ethnicity	*Composition: Aggregated r= -.02 [-.10, .06]*<br>*High tech r= na*<br>*Manufacture r=na*<br>*Service r= -.03 [-.14, .08]*<br>*Student r= -.02 [-.15, .10]*<br>*Composition: Heterogeneous r= -.06 [-.11, -.01]*<br>*High tech r= -.19 [-.34, -.04]*<br>*Manufacture r= -.05 [-.13, .04]*<br>*Service r= -.02 [-.13, .08]*<br>*Student r= -.04 [-.11, .03]*	*Industry type (see left column)*	Carter et al., 2019
Gender	*Composition: Heterogeneous r= -.03 [-.05, -.00]*<br>*High tech r= -.07 [-.14, -.00]*<br>*Manufacture r= -.11 [-.18, -.03]*<br>*Service r= -.03 [-.07, .02]*<br>*Student r= .02 [-.03, .07]*	*Industry type (see left column)*	Carter et al., 2019
Faultlines	*Composition: Heterogeneous r= -.05 [-.13, .02]*<br>*High tech r= na*<br>*Manufacture r= -.12 [-.19, -.12]*<br>*Service r= -.05 [-.11, .00]*<br>*Student r= -.11 [-.28, .05]*	*Industry type (see left column)*	Carter et al., 2019
Background diversity	*Innovation* ρ= -.13 [-.32, .05]<br>*Team innovation* ρ= -.10 [-.31, .11]	*Measurement level (see left column)*<br>*Measurement type*<br>*Independent rating* ρ= -.13 [-.33, .08]	Hulsheger et al., 2009
Gender	*Task performance r= -.01 [-.04, .02]*<br>*Gender egalitarianism*<br>*high r= .00 [-.03, .04]*	*Performance type (see below)*<br>*Culture (see left column and below)*	Schneid et al., 2015

	low r= -.07 [-.12, -.01]<br>*Humane orientation*<br>*high r= -.01 [-.04, .03]*<br>*low r= -.03 [-.09, .02]*<br>*Institutionalism collectivism*<br>*high r= -.05 [-.11. .00]*<br>*low r= .00 [-.04, .04]*<br>*Ingroup collectivism*<br>*high r= -.10 [-.18, -.02]*<br>*low r= .00 [-.04, .03]*<br>*Contextual performance r= -.10 [-.18, -.02]*<br><br>****Contextual performance** concerns aspects of an individual's performance, which maintains and enhances an organization's social network and the psychological climate that supports technical tasks (Motowidlo et al., 1997)*	*Objective r= -.02 [-.05, .02]*<br>*Gender egalitarianism*<br>*high r= .00 [-.04, .04]*<br>*low r= -.08 [-.15, -.01]*<br>*Humane orientation*<br>*high r= -.01 [-.06, .03]*<br>*low r= -.04 [-.10, .03]*<br>*Institutionalism collectivism*<br>*high r= -.03 [-.11, .04]*<br>*low r= -.02 [-.07, .02]*<br>*Ingroup collectivism*<br>*high r= -.15 [-.26, -.04]*<br>*low r= -.01 [-.04, .02]*<br>*Subjective r= -.01 [-.06, .03]*<br>*Gender egalitarianism*<br>*high r= .00 [-.05, .06]*<br>*low r= -.05 [-.14, .05]*<br>*Humane orientation*<br>*high r= -.01 [-.06, .04]*<br>*low r= -.02 [-.12, .09]*	

		Institutionalism collectivism<br>*high r= -.06 [-.13, .01]*<br>*low r= .02 [-.04, .08]*<br>*Ingroup collectivism*<br>*high r= .00 [-.05, .05]*<br>*low r= -.07 [-.18, .04]*	
Surface-level diversity	*Conflict mES= .08 [.00, .15]*<br>*Comm effectiveness mES= -.16 [-.32, .00]*<br>*Social integration mES= -.06 [-.14, .01]*	*See below section*	Stahl et al., 2009
Surface-level diversity	*Demographic diversity* ρ= -.07 [-.07, -.07]	*80%CI*	Stewart, 2006
Surface-level diversity	*Team performance*<br>*Age r= -.07*<br>*Sex r= -.05*<br>*Racial r= -.03*<br>*Faultlines r= -.14*<br>*Team satisfaction*<br>*Age r= -.09*<br>*Sex r= -.08*<br>*Racial r= -.02*<br>*Faultlines r= -.15*		Thatcher & Patel, 2011*(article retracted)
Demographic diversity	*Overall demographic diversity r= -.02 [-.04, .01]*<br>*Measurement*<br>*objective r= -.01 [-.03, .02]*<br>*subjective r= -.05 [-.08, -.03]*<br>*Rater type*<br>*member r= -.00 [-.05,.05]*<br>*internal leader r= .05 [-.03, .12]*<br>*external leader r= -.06 [-.09, -.03]*	Measurement type (see left column)<br>Task Complexity (see left column)<br>Rater type (see left column)<br>Performance type (see left column)	van Dijk et al., 2012

	Task complexity<br>*low r= -.03 [-.08, .03]*<br>*medium r= -.01 [-.04, .03]*<br>*high r= .01 [-.02, .04]*<br>*Performance type*<br>*in-role r= -.02 [-.04, -.01]*<br>*innovation r= .02 [-.02, .06]*		
Age	*Overall r= -.03 [-.07, .03]*<br>*Measurement*<br>*objective r= -.01 [-.04, .02]*<br>*subjective r= -.10 [-.13, -.07]*<br>*Rater type*<br>*member r= -.01 [-.11, .08]*<br>*internal leader r= .13 [.03, .22]*<br>*external leader r= -.14 [-.18, -.10]*<br>*Task complexity*<br>*low r= -.02 [-.09, .05]*<br>*medium r= -.01[-.07, .04]*<br>*high r= -.00 [-.04, .03]*<br>*Performance type*<br>*in-role r= -.04 [-.05, -.02]*<br>*innovation r= .00 [-.04,.04]*	Measurement type (see left column)<br>Task Complexity (see left column)<br>Rater type (see left column)<br>Performance type (see left column)	van Dijk et al., 2012
Ethnicity	*Overall r= -.05 [-11, .02]*<br>*Measurement*<br>*objective r= -.01 [-.06, .03]*<br>*subjective r= -.14 [-.17, -.11]*<br>*Rater type*<br>*member r= -.08 [-.16, .01]*	Measurement type (see left column)<br>Task Complexity (see left column)<br>Rater type (see left column)<br>Performance type (see left column)	van Dijk et al., 2012

	internal leader r=na<br>*external leader r= -.16 [-.20, -.12]*<br>*Task complexity*<br>*low r= -.03 [-.09, .03]*<br>*medium r= -.01 [-.06, .04]*<br>*high r= .17 [-.03, .35]*<br>*Performance type*<br>*in-role r= -.11 [-.14, -.08]*<br>*innovation r= -.07 [-.16, .02]*		
Gender	*Overall r= -.01 [-.05, .03]*<br>*Measurement*<br>*objective r= -.02 [-.06, .02]*<br>*subjective r= -.06 [-.08, -.03]*<br>*Rater type*<br>*member r= .04 [-.03, .10]*<br>*internal leader r= -.06 [-.16, .05]*<br>*external leader r= -.06 [-.09, -.02]*<br>*Task complexity*<br>*low r= -.04 [-.10, .01]*<br>*medium r= .01 [-.03, .05]*<br>*high r= -.04 [-.09, .02]*<br>*Performance type*<br>*in-role r= -.05 [-.07, -.02]*<br>*innovation r= -.01 [-.07, .06]*	Measurement type (see left column)<br>Task Complexity (see left column)<br>Rater type (see left column)<br>Performance type (see left column)	van Dijk et al., 2012
Nationality	*Overall r= -.01 [-.08, .07]*<br>*Measurement*<br>*objective r= -.08 [-.28, .13]*<br>*subjective r= .00 [-.07, .08]*	Measurement type (see left column)<br>Task Complexity (see left column)<br>Rater type (see left column)	van Dijk et al., 2012

	Rater type<br>*member r= -.03 [-.13, .07]*<br>*internal leader r= -.07 [-.22, .10]*<br>*external leader r= .04 [-.04, .12]*<br>*Task complexity*<br>*low r= na*<br>*medium r= -.01 [-.12, .10]*<br>*high r= .04 [-.07, .15]*<br>*Performance type*<br>*in-role r= .00 [-.07, .08]*<br>*innovation r= -.01 [-.16, .14]*	Performance type (see left column)	
Educational level	*Overall r= .00 [-.06, .07]*<br>*Measurement*<br>*objective r= .02 [-.03, .07]*<br>*subjective r= -.05 [-.08, -.01]*<br>*Rater type*<br>*member r= .02 [-.11, .15]*<br>*internal leader r= .21 [.09, .32]*<br>*external leader r= -.07 [-.12, -.02]*<br>*Task complexity*<br>*low r= na*<br>*medium r= -.14 [-.31, .05]*<br>*high r= .03 [-.02, .08]*<br>*Performance type*<br>*in-role r= -.04 [-.07, -.01]*<br>*innovation r= .20 [.11, .30]*	Measurement type (see left column)<br>Task Complexity (see left column)<br>Rater type (see left column)<br>Performance type (see left column)	van Dijk et al., 2012

Faultlines	*Faultlines r= -.06 [-.17, .05]*		van Dijk et al., 2012
Surface-level diversity	*Creativity/innovation* r_c*= -.02 [-.11, .06]*	<u>*Surface-level diversity*</u><br>*Team virtuality*<br>*Collocated* r_c*=.02 [-.07, .10]*<br>*Non-collocated* r_c*= -.16 [-.37, .04]*<br>*Task interdependence*<br>*Interdependent* r_c*= .00 [-.09, .10]*<br>*Independent* r_c*= -.10 [-.26, .06]*<br>*Task complexity*<br>*Complex* r_c*= .02 [-.07, .11]*<br>*Simple* r_c*= -.23 [-.33, -.12]*<br>*Task intellectiveness*<br>*Intellective* r_c*= -.04 [-.42, .34]*<br>*Judgemental* r_c*= -.02 [-.10, .06]*	Wang et al., 2019
Less job-related diversity	*Cohesion* ρ= -.03 [.10, -.16]<br>*Performance* ρ= -.07 [.00, -.15]	<u>*Less-job related diversity*</u><br>*Team type*<br>*TMTs* ρ= -07 [.07, -.22]<br>*Lower-level teams* ρ= -.07 [.02, -.16]	Webber & Donahue, 2001
Relation-oriented variety	*Innovation*<br>*Variety* ρ= -.087 [-.18, .01]<br>*Disparity* ρ= .013 [-.02, .05]<br>*Faultline strength* ρ= -.167 [-.30, -.03]	*Diversity operationalization (see left column)*	Wei et al., 2021
Social category diversity	*Team cognition-performance relationship*<br>*Homogeneous* ρ= .32 [.19, .45]<br>*Heterogeneous* ρ= .42 [.34, .50]	*Note that higher rho means team cogntition is more important to performance*	Niler et al., 2022

Faultlines	*Surface-level social faultlines*<br>*Social interaction quality* ρ= -.072 [-.14, -.01]<br>*Task interaction quality* ρ= -.09 [-.15, -.02]		Zhang & Chen, 2023

Table 2. Surface-level Attributes: Task-related

**Attribute**	**Performance**	**Moderators**	**Reference**
Ability	Composition (*homogeneous=heterogeneous*) Fisher's Z = -1.13, p > .05	<u>*Overall diversity*</u><br>Task difficulty<br>*Low* (*homogeneous>heterogeneous*) Fisher's Z= 1.85, p < .05<br>*Medium = ns*<br>*High* (*homogeneous<heterogeneous*) Fisher's Z= –2.37, p < .01<br><br>Task Type<br>Intellectual tasks (*homogeneous=heterogeneous*) Fisher's Z= –2.21, p = 0.99<br>Performance tasks (*homogeneous>heterogeneous*) Fisher's Z = 3.30, p < .01	Bowers et al., 2000
Task-related diversity	*Performance: Quality* ρ= .13 [.06, .19]<br>*Performance: Quantity* ρ= .07 [.01, .17]<br>*Social integration* ρ= -.04 [-.12, .03]	<u>*Team diversity-Social Integration*</u><br>Team size<br>Medium ρ= .02 [-.05, .11]<br>Small ρ= -.04 [-.12, .03]<br>Criterion report type<br>Manager/rater-reported ρ= -.19 [-.47, .08]<br>Self-report ρ= -.01 [-.05, .04]<br>Study setting<br>Organization ρ= -.02 [-.09, .02]<br>Laboratory ρ= -.03 [-.09, .06]	Horwitz & Horwitz, 2007
Task-oriented diversity	*Task-oriented diversity r = .04 [.02, .06]*<br>*Function r = .13 [.09, .17]*<br>*Education r = -.02 [-.06, .01]*<br>*Org. Tenure =.03 [-.01, .06]*	<u>*Task-oriented diversity*</u><br>Industry setting<br>*High-tech r = .06 [.04, .09]*<br>*Service r = -.00 [-.05, .05]*	Joshi & Roh, 2009

		Manufacturing r = .01 [-.05, .06]<br>*Interdependence*<br>*Low r = -.03 [-.11, .06]*<br>*Moderate r = .04 [.02, .06]*<br>*High r = .10 [.05, .15]*<br>*Team type*<br>*Short-term r = .08 [-.01, .16]*<br>*Long-term r = .04 [.02, .07]*<br>*Occupational gender demography*<br>*Maj. male setting r = .06 [.03, .09]*<br>*Balanced r = -.03 [-.08, .02]*<br>*Occupational race/ethnicity demography*<br>*Maj. white setting r = .04 [.02, .06]*<br>*Balanced r = -.02 [-.17, .13]*<br>*Occupational age demography*<br>*Maj. young setting r = .06 [.03, .09]*<br>*Balanced r = .01 [-.03, .04]*	
KSAs diversity	***Performance***<br>*Overall* ρ= .043 [-.29, .38]<br>***Process***<br>*Open Com.* ρ= .135 [-.16, .43]<br>*Freq. of Com.* ρ= .107 [-.31, .53]	<u>*KSA diversity*</u><br>*Uncertainty*<br>*Low* ρ= .078 [-.13, .28]<br>*High* ρ= .004 [-.41, .42]	Bui et al., 2019

Functional background	*Overall* ρ=.10 [.04, .15]<br>*Variety* ρ=.11 [.05, .15]<br>*-efficiency* ρ=.03 [-.05, .11]<br>*-general performance* ρ=.12 [.03, .18]<br>*-creativity/innovation* ρ=.18 [.02, .30]	Performance operationalization (see left column<br>Team Type<br>*-Design/cross-functional* ρ=.16 *[.08, .20]*<br>*-TMT* ρ=.07 [-.03, .16]<br>*-Other/mixed team type* ρ=-.01 [-.12, .10]	Bell et al., 2011
Educational background	*Variety* ρ= .01 [-.05, .08]<br>*-efficiency* ρ= -.02 [-.04, .01]<br>*-general performance* ρ= -.03 [-.08, .03]<br>*-creativity/innovation* ρ= .23 [.08, .33]	Performance operationalization (see left column<br>*-Team Type*<br>*-Design/cross-functional* ρ= .07 [-.06, .18]<br>*-TMT* ρ= .13 [.03, .21]<br>*-Other/mixed team type* ρ= -.05 [-.13, .04]	Bell et al., 2011
Education level	*Mean* ρ= .01 [-.08, .10]<br>*-intellectual teams* ρ= .11 [-.02, .21]<br>*-physical teams* ρ = *-.07 [one study]*<br>*Diversity* ρ= -.01 [-.05, .04]<br>*-variety* ρ= -.01 [-.09, .08]<br>*-other* ρ= -.01 [-.07, .06]	*Diversity conceptualization (see left column)*<br>*Team type (see left column)*	Bell et al., 2011
Org. tenure	*Mean* ρ= .08 [.01, .13]<br>*-efficiency* ρ= .14 [.09, .17]<br>*-general performance* ρ=.00 [-.11, .11]<br>*-creativity/innovation* ρ= *-.27[one study]*<br>*Diversity* ρ= .04 [-.01, .08]<br>*-separation* ρ= -.03 [-.08, .04]<br>*-variety* ρ= .06 [-.05, .16]	Performance operationalization (see left column<br>*Diversity conceptualization (see left column)*	Bell et al., 2011

	-disparity ρ= .04 [-.01, .10]		
Team tenure	*Mean* ρ= .09 [-.01, .18]<br>*-efficiency* ρ= .11 [.09, .17]<br>*-general performance* ρ= .02 [-.11, .11]<br>*-creativity/innovation* ρ= .10 [-.23, .40]<br>*Diversity* ρ= -.04 [-.10, .02]<br>*-disparity* ρ= -.04 [-.10, .01]	Performance operationalization (see left column<br>*Diversity conceptualization (see left column)*	Bell et al., 2011
Education	*Composition: Aggregated r= -.03 [-.10, .05]*<br>*High tech r= -.10 [-.29, .09]*<br>*Manufacture r= .08 [-.28, .43]*<br>*Service r= .03 [-.08, .13]*<br>*Student r= -.13 [-.26, .03]*<br>*Composition: Heterogeneous r= -.03 [-.08, .01]*<br>*High tech r= -.11 [-.15, -.07]*<br>*Manufacture r= na*<br>*Service r= .06 [-.05, .16]*<br>*Student r= .03 [-.12, .18]*	Industry type (see left column)	Carter et al., 2019
Tenure	*Composition: Aggregated r= .09 [.05, .13]*<br>*High tech r= .03 [-.02, .08]*<br>*Manufacture r= .17 [.10, .23]*<br>*Service r= .05 [.02, .09]*<br>*Student r= .17 [.03, .31]*<br>*Composition: Heterogeneous r= -.00 [-.05, .04]*<br>*High tech r= .08 [.01, .15]*<br>*Manufacture r= .00 [-.07, .08]*	Industry type (see left column)	Carter et al., 2019

	Service r= -.03 [-.10, .03]<br>*Student r= .05 [-.15, .25]*		
Familiarity	*Composition: Aggregated r= .04 [-.02, .11]*<br>*High tech r= .15 [.09, .21]*<br>*Manufacture r= .17 [.08, .26]*<br>*Service r= -.08 [-.16, .00]*<br>*Student r= -.10 [-.18, -.01]*	Industry type (see left column)	Carter et al., 2019
Functional Background	*Composition: Aggregated r= .04 [-.01, .09]*<br>*High tech r= .10 [.04, .17]*<br>*Manufacture r= .42 [.12, .73]*<br>*Service r= -.03 [-.13, .07]*<br>*Student r= .07 [-.01, .15]*<br>*Composition: Heterogeneous r= .06 [.03, .09]*<br>*High tech r= .12 [.10, .15]*<br>*Manufacture r= .07 [-.07, .21]*<br>*Service r= .04 [-.02, .09]*<br>*Student r= -.01 [-.08, .06]*	Industry type (see left column)	Carter et al., 2019
Team Tenure	*Additive*<br>*Performance* ρ= .20 [.15, .24]<br>*Process*<br>*Cognition* ρ= .12 [.00, .23]<br>*Motivational-affective* ρ= .01 [-.03, .05]<br>*Behavioral* ρ= .06 [.03, .10]<br>*Collective*	<u>*Supplemental analyses*</u><br>*Interdependence*<br>*Additive-Performance*<br>*Low* ρ= .11 [.04, .17]<br>*Med* ρ= .07 [.01, .13]<br>*High* ρ= .29 [.23, .35]<br>*Additive-Cognition*	Gonzalez-Mulé et al., 2020

	Performance ρ= .11 [.05, .16]<br>*Process*<br>*Cognition* ρ= .07 [-.04, .18]<br>*Motivational-affective* ρ= .10 [-.02, .21]<br>*Behavioral* ρ= -.02 [-.06, .02]<br>*Dispersion*<br>*Performance* ρ= .08 [.03, .14]<br>*Process*<br>*Cognition* ρ= .05 [-.01, .11]<br>*Motivational-affective* ρ= .02 [-.05, .09]<br>*Behavioral* ρ= .10 [.03, .17]	*Low* ρ= -.06 [-.23, .11]<br>*Med* ρ= .16 [.07, .25]<br>*High* ρ= .28 [-.15, .70]<br>*Level of Specificity*<br>*Additive-Performance*<br>*Job* ρ= .41 [.35, .47]<br>*Team* ρ= .16 [.12, .22]<br>*Org.* ρ= .10 [.03, .18]<br>*Additive-Cognition*<br>*Job* ρ= .09 [.01, .17]<br>*Team* ρ= .28 [.13, .48]<br>*Org.* ρ= .03 [-.09, .16]<br>*Performance Outcome*<br>*Additive-Performance*<br>*Objective* ρ= .30 [.23, .37]<br>*Subjective* ρ= .07 [.04, .12]	
Job-relevant diversity	*Innovation* ρ= .155 [.00, .31]<br>*Team innovation* ρ= .24 [.04, .44]	Measurement level (see left column)<br>Measurement method<br>*Self-rating* ρ= -.01 [-.16, .14]<br>*Independent rating* ρ= .16 [.02, .30]	Hulsheger et al., 2009
Team longevity	*Innovation* ρ= .02 [-.14, .18]<br>*Team innovation* ρ= -.06 [-.26, .14]	Measurement level (see left column)<br>Measurement method<br>*Self-rating* ρ= -.37 [-.50, -.24]<br>*Independent rating* ρ= .13 [.02, .24]	Hulsheger et al., 2009
Entrepreneurial	*Aggregated Composition r= .14 [.10, .18]*	Industry type (see left column)	Jin et al., 2016

Team Composition - Mixed	*High-tech r= .08 [.03, .14]*<br>*Low-tech r= .21 [.16, .25]*<br>*Heterogeneity r= .05 [.01, .09]*<br>*High-tech r= .07 [.03, .11]*<br>*Low-tech r= -.03 [-.11, .06]*		
Surface-level diversity	*Aggregated*<br>*Expertise* ρ= .16 [.16, .16]<br>Heterogeneity<br>Expertise ρ= -.05 [-.05, -.05]<br>Organizational tenure ρ= -.08 [-.25, .10]	*80%CI*	Stewart, 2006
Surface-level diversity	*Team performance*<br>*Functional r= -.07*<br>*Educational r= -.04*<br>*Tenure r= -.06*<br>*Team satisfaction*<br>*Functional r= -.03*<br>*Educational r= -.05*<br>*Tenure r= -.03*		Thatcher & Patel, 2011*(article retracted)
Job-related diversity	*Job-related diversity r= .05 [.01, .08]*<br>*Measurement*<br>*objective r= .02 [-.01, .06]*<br>*subjective r= .04 [.01, .07]*<br>*Rater type*<br>*member r= .02 [-.06, .09]*<br>*internal leader r= .03 [-.04, .10]*<br>*external leader r= .09 [.06, .13]*<br>*Task complexity*<br>*low r= -.04 [-.09, .02]*<br>*medium r=.03[-.02, .09]*	Measurement type (see left column)<br>Task Complexity (see left column)<br>Rater type (see left column)<br>Performance type (see left column)	van Dijk et al., 2012

	high r=.06 [.02, .09]<br>*Performance type*<br>*in-role r= .04 [.02, .06]*<br>*innovation r= .09 [.04, .14]*		
Functional background	*Functional background r= .07 [.03, .12]*<br>*Measurement*<br>*objective r= .06 [.01, .10]*<br>*subjective r= .12 [.09, .16]*<br>*Rater type*<br>*member r= .13 [.02, .24]*<br>*internal leader r= .15 [.04, .25]*<br>*external leader r= .13 [.09, .17]*<br>*Task complexity*<br>*low r= na*<br>*medium r= .04 [-.04, .12]*<br>*high r= .08 [.04, .12]*<br>*Performance type*<br>*in-role r= .10 [.07, .13]*<br>*innovation r= .13 [.06, .19]*	Measurement type (see left column)<br>Task Complexity (see left column)<br>Rater type (see left column)<br>Performance type (see left column)	van Dijk et al., 2012
Educational background	*Educational background r= -.00 [-.05, .05]*<br>*Measurement*<br>*objective r= -.00 [-.06, .05]*<br>*subjective r= -.00 [-.09, .08]*<br>*Rater type*<br>*member r= .02 [-.16, .19]*<br>*internal leader r= na*	Measurement type (see left column)<br>Task Complexity (see left column)<br>Rater type (see left column)<br>Performance type (see left column)	van Dijk et al., 2012

	external leader r= .06 [-.06, .17]<br>*Task complexity*<br>*low r= na*<br>*medium r= -.01 [-.18, .16]*<br>*high r= .02 [-.05, .09]*<br>*Performance type*<br>*in-role r= -.00 [-.05, .05]*<br>*innovation r= -.00 [-.16, .16]*		
Tenure	*Tenure r= -.01 [-.05, .04]*<br>*Org. tenure r= -.00 [-.07, .06]*<br>*Team tenure r= -.02 [-.07, .04]*	*Measurement type*<br>*objective r= -.01 [-.05, .03]*<br>*subjective r= .01 [-.02, .04]*<br>*Rater type*<br>*member r= -.06 [-.15, .04]*<br>*internal leader r= .00 [-.08, .07]*<br>*external leader r= .08 [.04, .12]*<br>*Task complexity*<br>*low r= -.04 [-.10, .02]*<br>*medium r= -.05 [-.12, .02]*<br>*high r= .01 [-.03, .05]*<br>*Performance type*<br>*in-role r= .01 [-.02, .03]*<br>*innovation r= .03 [-.03, .09]*	van Dijk et al., 2012
Job-related diversity	*Cohesion* ρ= .10 [-.02, .23]<br>*Performance* ρ= .02 [-.03, .07]	*<u>Highly-job related diversity</u>*<br>*Team type*<br>*TMTs* ρ= .03 [-.02, .08]<br>*Lower-level teams* ρ= -.09 [-.22, .04]	Webber & Donahue, 2001
Task-oriented variety *(innovation)*	*Innovation*<br>*Variety* ρ= .11 [.07, .14]<br>*Disparity* ρ= .08 [.04, .12]<br>*Faultline strength* ρ= .11 [.00, .22]	*Diversity operationalization (see left column)*	Wei et al., 2021

Job-related diversity *(creativity and innovation)*	<u>*Creativity/innovation*</u><br>*Job-related diversity* ρ= .05 [.01, .09]<br>*Functional background* ρ= .06 [.01, .11]<br>*Educational background* ρ= .09 [.01, .16]<br>*Team tenure* ρ= .06 [-.02, .14]	<u>*Supplemental Analyses*</u><br>*Country culture*<br>*Collectivistic* ρ= *.04 [-.01, .09]*<br>*Individualistic* ρ= *.07 [.00, .13]*	Byron et al., 2022
Functional diversity	*Team cognition-performance relationship*<br>*Homogeneous* ρ= .36 [.17, .56]<br>*Heterogeneous* ρ= .29 [.18, .39]	*Note that higher rho means team cogntition is more important to performance*	Niler et al., 2022
Faultlines	*Surface-level task faultlines*<br>*Social interaction quality* ρ= .08 [-.06, .24]<br>*Task interaction quality* ρ= .155 [.07, .28]		Zhang & Chen, 2023

Table 3. Deep-level Attributes

Attribute	Performance	Moderators	Reference
Personality	Composition<br>(*homogeneous=heterogeneous*)<br>Fisher's Z = -1.2, p > .05	<u>*Overall diversity*</u><br>Task difficulty<br>*Low*<br>(*homogeneous>heterogeneous*)<br>Fisher's Z= 1.85, p < .05<br>*Medium = ns*<br>*High*<br>(*homogeneous<heterogeneous*)<br>Fisher's Z= –2.37, p < .01<br><br>Task Type<br>Intellectual tasks<br>(*homogeneous=heterogeneous*)<br>Fisher's Z= –2.21, p = 0.99<br>Performance tasks<br>(*homogeneous>heterogeneous*)<br>Fisher's Z = 3.30, p < .01	Bowers et al., 2000
Personality<br>*Conscientiousness*	 *Overall* ρ=.11 [.04, .14]<br>*mean* ρ=.14 [.05, .18]<br>*maximum* ρ=.09 [-.02, .18]<br>*minimum* ρ=.12 [.00, .20]<br>*heterogeneity* ρ=.-.03 [-.12, .06]	 Statistical operationalizations (see left column)<br>Study setting<br>Lab ρ= .04 [-.02, .08]<br>Field ρ= .30 [.17, .31]	Bell, 2007
Agreeableness	*Overall* ρ=.12 [.04, .16]<br>*mean* ρ=.17 [.06, .21]<br>*maximum* ρ=.09 [-.04, .18]<br>*minimum* ρ=.19 [.04, .27]<br>*heterogeneity* ρ=.-.04 [-.09, .03]	Statistical operationalizations (see left column)<br>Study setting<br>Lab ρ=.03 [-.03, .08]<br>Field ρ= .31 [.16, .34]	Bell, 2007
Extraversion	*Overall* ρ=.09 [.03, .11]<br>*mean* ρ=.10 [.03, .13]<br>*maximum* ρ=.11 [-.01, .18]<br>*minimum* ρ=.05 [-.04, .12]<br>*heterogeneity* ρ=.03 [-.02, 07]	Statistical operationalizations (see left column)<br>Study setting<br>Lab ρ=.06 [.00, .10]<br>Field ρ= .15 [.06, .19]	Bell, 2007

Emotional Stability	*Overall* ρ=.04 [-.02, .09]<br>*mean* ρ=.13 [.05, .16]<br>*maximum* ρ=.13 [-.03, .25]<br>*minimum* ρ=.07 [-.02, .13]<br>*heterogeneity* ρ=.02 [-.07, .10]	Statistical operationalizations (see left column)<br>Study setting<br>Lab ρ=.03 [-.04, .09]<br>Field ρ= .06 [-.05, .15]	Bell, 2007
Openness to Experience	*Overall* ρ=.05 [-.02,.10]<br>*mean* ρ=.11 [.02, .16]<br>*maximum* ρ=.10 [.01, .15]<br>*minimum* ρ=.05 [-.06, .15]<br>*heterogeneity* ρ= -.03 [-.10, .06]	Statistical operationalizations (see left column)<br>Study setting<br>Lab ρ=.00 [-.06, .07]<br>Field ρ= .20 [.05, .27]	Bell, 2007
Collectivism	*Overall* ρ=.25 [.09, .31]<br>*mean or sum* ρ=.31 [.10, .38]<br>*heterogeneity* ρ=.02 [-.11, .14; lab only]	Statistical operationalizations (see left column)<br>Study setting<br>Lab ρ=.00 [-.09, .08]<br>Field ρ= .35 [.12, .47]	Bell, 2007
Preference for Teamwork	*Overall* ρ=.18 [.02, .29]<br>*mean* ρ=.23 [.08, .32]<br>*heterogeneity* ρ=.01 [-.29, .31; lab only]	Statistical operationalizations (see left column)<br>Study setting<br>Lab ρ=.01 [-.26, .27]<br>Field ρ= .22 [.07, .30]	
Emotional Intelligence	*Mean only* ρ=.18 [.06, .26]	Statistical operationalizations (see left column)<br>Study setting<br>Lab ρ=.20 [.09, .26]<br>Field ρ= .10 [-.22, .39]	Bell, 2007
GMA	*Overall* ρ=.27 [.17, .29]<br>*mean or sum* ρ=.31 [.20, .31]<br>*maximum* ρ=.27 [.07, .37]<br>*minimum* ρ=.34 [.13, .42]<br>*heterogeneity* ρ=.01 [-.07, .09]	Statistical operationalizations (see left column)<br>Study setting<br>Lab ρ=.31 [.19, .29]<br>Field ρ= .18 [.04, .25]	Bell,2007
Personality<br>*Agreeableness*	*Composition: Aggregated r= .14 [.05, .24]*<br>*High tech r= .15 [-.06, .36]*	*Industry type (see left column)*	Carter et al., 2019

	Manufacture r=na<br>*Service r= .40 [.17, .63]*<br>*Student r= .12 [.01, .22]*<br>*Composition: Heterogeneous r= -.14 [-.28, .00]*<br>*High tech r=na*<br>*Manufacture r=na*<br>*Service r= -.24 [-.58, .10]*<br>*Student r= -.12 [-.28, .03]*		
Conscientiousness	*Composition: Aggregated r= .08 [-.00, .16]*<br>*High tech r= na*<br>*Manufacture r=na*<br>*Service r= .15 [.02, .29]*<br>*Student r= .02 [-.09, .12]*<br>*Composition: Heterogeneous r= -.12 [-.25, .01]*<br>*High tech r=na*<br>*Manufacture r=na*<br>*Service r= -.12 [-.29, .05]*<br>*Student r= -.11 [-.50, .28]*	*Industry type (see left column)*	Carter et al., 2019
Extraversion	*Composition: Aggregated r= .10 [.01, .18]*<br>*High tech r=na*<br>*Manufacture r=na*<br>*Service r= .04 [-.09, .17]*<br>*Student r= .13 [.02, .23]*<br>*Composition: Heterogeneous r= .04 [-.10, .18]*<br>*High tech r=na*<br>*Manufacture r=na*<br>*Service r= .04 [-.10, .18]*<br>*Student r=na*	*Industry type (see left column)*	Carter et al., 2019
Openness to Experience	*Composition: Aggregated r= .13 [.02, .25]*	*Industry type (see left column)*	Carter et al., 2019

	High tech r=na<br>*Manufacture r=na*<br>*Service r= .09 [.01, .26]*<br>*Student r= .14 [.02, .24]*<br>*Composition: Heterogeneous r= .14 [-.15, .44]*<br>*High tech r=na*<br>*Manufacture r=na*<br>*Service r=na*<br>*Student r= .14 [-.15, .44]*		
Emotional Stability	*Composition: Aggregated r= .13 [.02, .24]*<br>*High tech r= na*<br>*Manufacture r=na*<br>*Service r= - .00 [-.36, .36]*<br>*Student r= .14 [.02, .27]*<br>*Composition: Heterogeneous r=na*<br>*High tech r=na*<br>*Manufacture r=na*<br>*Service r=na*<br>*Student r=na*	*Industry type (see left column)*	Carter et al., 2019
Cognitive Ability	*Composition: Aggregated r= .07 [-.00, .14]*<br>*High tech r= -.01 [-.19, .17]*<br>*Manufacture r=na*<br>*Service r= .22 [.01, .43]*<br>*Student r= .09 [.02, .16]*<br>*Composition: Heterogeneous r= .07 [-.03, .18]*<br>*High tech r= -.20 [-.42, .02]*<br>*Manufacture r= na*<br>*Service r= .01 [-.19, .21]*<br>*Student r= .10 [.01, .19]*	*Industry type (see left column)*	Carter et al., 2019

Deep-level cultural diversity	*Conflict mES= .05 [-.07, .16]*<br>*Comm effectiveness mES= .14 [-.04, .32]*<br>*Social integration mES= .00 [-.10, .11]*		Stahl et al., 2009
Mixed-cultural diversity *(Surface-level and Deep-level)*	*Creativity mES= .16 [.00, .32]*<br>*Conflict mES= .07 [.01, .13]*<br>*Task conflict mES= .10 [.02, .18]*<br>*Relationship conflict mES= .05 [.03, .13]*<br>*Process conflict mES= .01 [.11, .14]*<br>*Communication effectiveness mES= -.03 [-.15, .09]*<br>*Satisfaction mES= .15 [.05, .25]*<br>*Social integration mES= -.07 [-.12, -.02]*<br>*Performance mES= -.02 [-.04, .00]*	*Task complexity*<br>*-Conflict*<br>*Low mES= -.10 [-.24, .04]*<br>*High mES= .09 [.01, .17]*<br>*Team size*<br>*-Conflict*<br>*Small mES= .12 [.03, .21]*<br>*Large mES= .08 [-.02, .17]*<br>*-Comm effectiveness*<br>*Small mES= .14 [-.07, .36]*<br>*Large mES= -.27 [-.45, -.09]*<br>*-Satisfaction*<br>*Small mES= .28 [.10, .46]*<br>*Large mES= -.04 [-.20, .12]*<br>*-Social integration*<br>*Small mES= -.13 [-.09, .02]*<br>*Large mES= -.17 [-.27, .07]*<br>*Geographic dispersion*<br>*-Conflict*<br>*Collocated mES= .10 [.04, .16]*<br>*Dispersed mES= -.14 [-.32, -.04]*<br>*-Social integration*<br>*Collocated mES= -.08 [-.14, -.02]*<br>*Dispersed mES= .11 [-.03, .25]*	Stahl et al., 2009

		Team tenure<br>*-Conflict*<br>*≤20h mES= .00 [-.10, .10]*<br>*>20h mES= .12 [.05, .20]*<br>*-Comm effectiveness*<br>*≤20h mES= .12[-.06, .29]*<br>*>20h mES= -.14 [-.30, .02]*<br>*-Satisfaction*<br>*≤20h mES= .19 [.07, .30]*<br>*>20h mES=.02 [-.18, .22]*<br>*-Social integration*<br>*≤20h mES= -.12 [-.23, .00]*<br>*>20h mES= -.07 [-.13, -.01]*	
Deep-level diversity	*Overall Deep-level diversity r= -.01 [-.06, .03]*<br>*Personality r= .04 [-.02, .10]*<br>*Extraversion r= .05 [-.05, .16]*<br>*Agreeableness r= -.03 [-.14, .07]*<br>*Conscientiousness r= -.09 [-.19, .01]*<br>*Neuroticism r= .04 [-.09, .17]*<br>*Openness r= .15 [-.00, .30]*<br>*Value r= -.07 [-.18, .04]*<br>*Cognitive r= -.06 [-.16, .03]*<br>*Attitude r= -.04 [-.14, .06]*<br>*Ability r= -.09 [-.29, .13]*	<u>*Deep-level diversity*</u><br>*Measurement*<br>*objective r= -.01 [-.07, .05]*<br>*subjective r= .00 [-.04, .05]*<br>*Rater type*<br>*member r= -.07 [-.15, .02]*<br>*internal leader r= .01 [-.12. .14]*<br>*external leader r= .01 [-.04, .06]*<br>*Task complexity*<br>*low r= .03 [-.06, .13]*<br>*medium r= .03 [-.02, .08]*<br>*high r= -.18 [-.28, -.08]*<br>*Performance type*<br>*in-role r= -.01 [-.05, .03]*<br>*innovation r= .10 [-.01, .20]*	van Dijk et al., 2012

Deep-level diversity	*Creativity/innovation* r_c*= .16 [.06, .27]*	<u>*Deep-level diversity*</u><br>*Team virtuality*<br>*Collocated* r_c*= .18 [.07, .31]*<br>*Non-collocated* r_c*= .02 [-.03, .06]*<br>*Task interdependence*<br>*Interdependent* r_c*= .19 [.10, .30]*<br>*Independent* r_c*= -.10 [-.43, .23]*<br>*Task complexity*<br>*Complex* r_c*= .16 [.06, .28]*<br>*Simple* r_c*= .05 [-.14, .24]*<br>*Task intellectiveness*<br>*Intellective* r_c*= .09 [-.04, .22]*<br>*Judgemental* r_c*= .16 [.03, .32]*	Wang et al., 2019
Deep-level diversity	*Task performance r= -.01 [-.03, .01]*<br>*Emergent states r= -.07 [-.10, -.05]*<br>*Task complexity*<br>*Low r= -.00 [-.05, .04]*<br>*High r= -.07 [-.11, -.03]*<br>*Team type*<br>*Exexcutive r= -.22 [-.30, -.14]*<br>*Non-executive r= -.06 [-.09, -.03]*<br>*Team process r= -.10 [-.14, -.07]*<br>*Task complexity*<br>*Low r= -.01 [-.08, .07]*<br>*High r= -.14 [-.19,-.10]*	*Task complexity (see left column)*<br>*Team type (see left column)*	Triana et al., 2021

	Team type<br>*Exexcutive r= -.30 [-.36, -.24]*<br>*Non-executive r= -.02 [-.06, .02]*<br>*Team conflict r= .12 [.07, .16]*<br>*Task complexity*<br>*Low r= .18 [.09, .28]*<br>*High r= .13 [.07, .19]*<br>*Team type*<br>*Exexcutive r= .24 [.13, .35]*<br>*Non-executive r= .09 [.04, .15]*		
Personality diversity	*Emergent states r= -.06 [-.10, -.02]*<br>*Team process r= .03 [-.03, .08]*<br>*Team conflict r= .05 [-.01, .12]*		Triana et al., 2021
Values diversity	*Emergent states r= -.10 [-.14, -.06]*<br>*Team process r= -.26 [-.31, -.21]*<br>*Team conflict r= .16 [.14, .34]*		Triana et al., 2021
Cultural diversity	*Emergent states r= -.02 [-.10, .06]*<br>*Team process r= .03 [-.07, .13]*<br>*Team conflict r= .24 [.14, .34]*		Triana et al., 2021
Deep-level diversity	*Aggreagated*<br>*Personality* ρ= .26 [.12, .41]<br>*Cognitive ability* ρ= .40 [.31, .50]	*80%CI*	Stewart, 2006
Personality	*Elevated [90% CI]*<br>*Extraversion* ρ= .04 [-.05,.13]<br>*Agreeableness* ρ= .24 [.09, .30]	<u>*Team Type*</u><br>*Elevated*<br>*Agreeableness*<br>*Professional* ρ= .51 [.42,.61]	Peeters et al., 2005

	Conscientiousness ρ= .20 [09, .31]<br>*Emotional Stability* ρ= .04 [-.06, .13]<br>*Openness* ρ= .03 [-.14, .20]<br>*Variability*<br>*Extraversion* ρ= .06 [-.06, .18]<br>*Agreeableness* ρ= -.12 [-.16,-.07]<br>*Conscientiousness* ρ= -.24 [-.33, -.14<br>*Emotional Stability* ρ= .02 [-.13, .16<br>*Openness* ρ= -.01 [-.15, .12]	*Student* ρ= .02 [-.11, .15]<br>*Conscientiousness*<br>*Professional* ρ= .42 [.33. .51]<br>*Student* ρ= .00 [-.07, .07]<br>*Emotional Stability*<br>*Professional* ρ= .14 [-.05, .32]<br>*Student* ρ= -.04 [-.07, -.01]<br>*Variability*<br>*Agreeableness*<br>*Professional* ρ= -.13 [-.16, -.11]<br>*Student* ρ= -.08 [-.15, -.01]<br>*Conscientiousness*<br>*Professional* ρ= -.21 [-.34, -.08]<br>*Student* ρ= -.22 [-.36, -.08]<br>*Emotional Stability*<br>*Professional* ρ= .16 [-.01, .33]<br>*Student* ρ= -.11 [-.20, -.02]<br>*Openness*<br>*Professional* ρ= -.11 [-.14, -.08]<br>*Student* ρ= .08 [-11, .26]	
General mental ability	*Operationalization*<br>*Average r = .29 [.23, .36]*<br>*High-member r = .21 [.14, .28]*<br>*Low-member r = .25 [.17, .33]*<br>*Standard Deviation r = -.03 [-.09, .03]*	<u>*General mental ability*</u><br>*Study-setting*<br>*Lab r = .37 [.32, .42]*<br>*Field r = .14 [.01, .26]*	Devine & Phillips, 2001

Team Orientation	*Team performance* ρ= .46 [.31, .60]<br>*Individual Performance* ρ= -.45 [-.67, -.23]<br>*Conflict* ρ= -.37 [-.49, -.25]<br>*Satisfaction* ρ= .37 [.28, .46]<br>*Innovation/learning* ρ= .84 [.58, 1.09]<br>*Cohesion* ρ= *60 [.44, 76]*<br>*Process*<br>*Communication* ρ= .82 [.65, 1.01]<br>*Coordination* ρ= .78 [.63, 1.01]<br>*Cooperation* ρ= .73 [.63, 1.03]<br>*Trust* ρ= .60 [.41, .79]<br>*Shared mental models* ρ= .69 [.45, .94]<br>*Backup behaviors* ρ= *.72 [.49, .94]*	<u>*Team orientation-team peformance*</u><br>*Type of team orientation*<br>*Individual-level team orientation*<br>ρ= .23 [.14, .31]<br>*Team-level team orientation*<br>ρ= .53 [.35, .71]<br>*Type of team*<br>*Student* ρ= .20 [.00, .39]<br>*Employee* ρ= .56 [.42, 70]	Kilcullen et al., 2022
Faultlines	*Deep-level social faultlines*<br>*Social interaction quality* ρ= -.13 [-.24, -.03]<br>*Task interaction quality* ρ= -.25 [-.39, -.10]<br>*Deep-level task faultlines*<br>*Social interaction quality* ρ= -.25 [-.35, -.16]<br>*Task interaction quality* ρ= -.28 [-.40, -.16]		Zhang & Chen, 2023
